# Probing the Antiaromaticity and Coordination Chemistry of Bowl-Shaped Zinc(II) Norcorrole

**DOI:** 10.1021/acs.inorgchem.4c01146

**Published:** 2024-05-23

**Authors:** David Bradley, Ruoming Tian, Mohan M. Bhadbhade, Lauren K. Macreadie, Chowdhury Hasan Sarowar, Martin D. Peeks

**Affiliations:** † School of Chemistry, UNSW Sydney, New South Wales 2052, Australia; ‡ Mark Wainwright Analytical Centre, UNSW Sydney, New South Wales 2052, Australia; § Bioanalytical Mass Spectrometry Facility, Mark Wainwright Analytical Centre, UNSW Sydney, New South Wales 2052, Australia

## Abstract

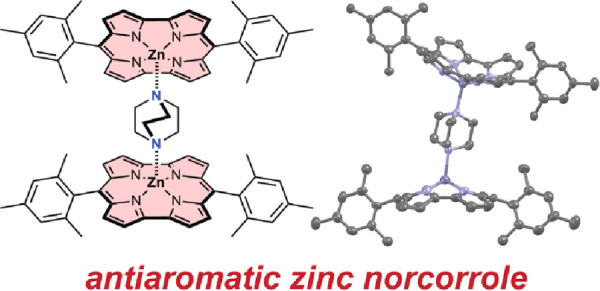

Zinc norcorrole was prepared as its pyridine complex (ZnNc·pyridine) by metalation of freebase norcorrole. The ZnNc·pyridine complex is distinctly bowl-shaped, as demonstrated by both X-ray crystallography and nuclear magnetic resonance (NMR) spectroscopy. NMR spectroscopy showed characteristic ring current deshielding effects, with different magnitudes on either face of the bowl-shaped complex. Exchanging the pyridine ligand with the bidentate ligand DABCO results in the formation of a stable (ZnNc)_2_·DABCO sandwich complex, which was also characterized by crystallography and NMR spectroscopy. The NMR resonances of the axial ligands in all of the complexes demonstrate that the paratropic ring current in zinc norcorrole is approximately 40 nA/T, which is comparable in magnitude to the diatropic ring current in porphyrin. Analysis of the ligand-exchange processes on addition of DABCO to ZnNc·pyridine showed that ZnNc coordinates to axial nitrogen-containing ligands with approximately 1000-fold higher binding constants than analogous zinc porphyrins.

## Abstract 2

Norcorrole is an antiaromatic tetrapyrrole; it is the antiaromatic analogue of famously aromatic porphyrin. We report the preparation of zinc norcorrole and its coordination complexes. Zinc norcorrole is extremely curved and binds ligands much more strongly than aromatic zinc porphyrin. The ring current strength of zinc norcorrole is almost the same as that in zinc porphyrin, albeit paratropic.

Aromatic molecules such as metalloporphyrins are common structural motifs in supramolecular structures. When π-conjugated building blocks are connected, emergent properties can arise such as macrocyclic aromaticity,^1,2^ low band gaps,^3^ and long-wavelength absorption. While aromatic compounds impart stability to such structures, the small HOMO–LUMO gaps of antiaromatic compounds could offer advantages in areas such as single molecule conductance^4^ and energy storage.^5^ Moreover, the recent development of synthetic methods to prepare antiaromatic compounds has challenged the notion that such compounds are inherently unstable.^6^ Nickel norcorrole^6^ has proven a versatile compound for exploring the properties of antiaromaticity, demonstrating increased conductance^7^ (compared to an aromatic analogue) and through-space interactions.^8^

To explore the antiaromaticity of norcorrole beyond its local environment, synthetic modifications must be made to enable the use of covalent or noncovalent interactions. The Nitschke group used norcorrole to construct the faces of an “antiaromatic-walled nanospace”; encapsulated molecules showed deshielded NMR spectra owing to the proximity of the norcorrole fragments.^9^ One strategy to build supramolecular complexes with metalloporphyrins involves binding ligands to a sufficiently Lewis-acidic metal center. The binding constant of pyridine to zinc porphyrins is on the order of 10^4^ M^–1^,^10−12^ and porphyrins have been used to construct supramolecular rings and ladders, often taking advantage of cooperativity.^13,14^ Given the structural similarities between norcorrole and porphyrin, we might expect that similar binding modes with norcorrole subunits are possible, allowing for the construction of exotic antiaromatic supramolecular structures.

Both the Shinokubo^15^ and Tokitoh^16^ groups independently reported aromatic phosphorus norcorroles, comprising methoxy-substituted and phenyl-substituted P(V) cores, respectively. In both cases, the NMR spectra of the molecule show that resonances corresponding to the peripheral substituents are shielded due to the diatropic ring current of the doubly reduced 18π norcorrole ligand. However, the binding of ligands to the central metals of antiaromatic norcorroles has not yet been explored. Analogous to porphyrin chemistry, we suspect that the Zn^2+^ ion in a zinc norcorrole (ZnNc) will coordinate to Lewis basic ligands, forming five-coordinate complexes. In these cases, successful coordination of a ligand should be evidenced by a characteristic NMR deshielding of resonances corresponding to the ligand.

In 2017, the Shinokubo group developed a protocol for the synthesis of a freebase norcorrole (H_2_Nc) and demonstrated that it could be metalated with Pd^2+^.^17^ The large Pd^2+^ ion rendered the norcorrole appreciably nonplanar, but despite its bowl-shaped geometry, palladium norcorrole (PdNc) exhibited strong signs of antiaromaticity. Similar properties were observed in the case of platinum norcorrole (PtNc).^18^ More generally, curved π systems have attracted interest for their unique binding^19^ and their electronic properties.^20,21^

In our initial attempts to synthesize ZnNc, we used standard zinc porphyrin metalation conditions (i.e., CHCl_3_, MeOH, Zn(OAc)_2_, temperatures from room temperature to reflux), but there was no evidence of metalation. It has previously been shown that corrole, another ring contracted porphyrinoid, can be metalated in refluxing pyridine.^22^ Reacting H_2_Nc under these conditions (Figure 1a) resulted in the formation of a new product evidenced by the absence of internal H_2_Nc N–H protons in the ^1^H NMR spectrum (SI Figure S1). The insertion of Zn^2+^ into the norcorrole core seemingly disrupts the symmetry of the norcorrole—while H_2_Nc has five unique proton environments (not including internal N–H protons), the analogous ZnNc structure has seven proton environments (SI Figure S1). NOE experiments reveal two distinct *o*-CH_3_ and Ar–H environments at room temperature, suggesting that ZnNc has a bowl-shaped geometry. Similar spectra have been reported for PdNc at a low temperature.^17^ In ZnNc, the curvature results in a 2.36 ppm difference in chemical shift between *o*-CH_3_ protons on the concave and convex face of ZnNc. This bowl-shaped geometry is surprising considering that the crystal structure of CuNc is planar,^17^ and Cu^2+^ has a comparable ionic radius to Zn^2+^,^23^ although it is possible that the pyridine ligand contributes to the distortion by withdrawing the zinc atom out of the plane of the norcorrole. With pyridine-*d*_5_ as the reaction solvent, the pyridine ligand is not visible by ^1^H NMR spectroscopy.

**Figure 1:**
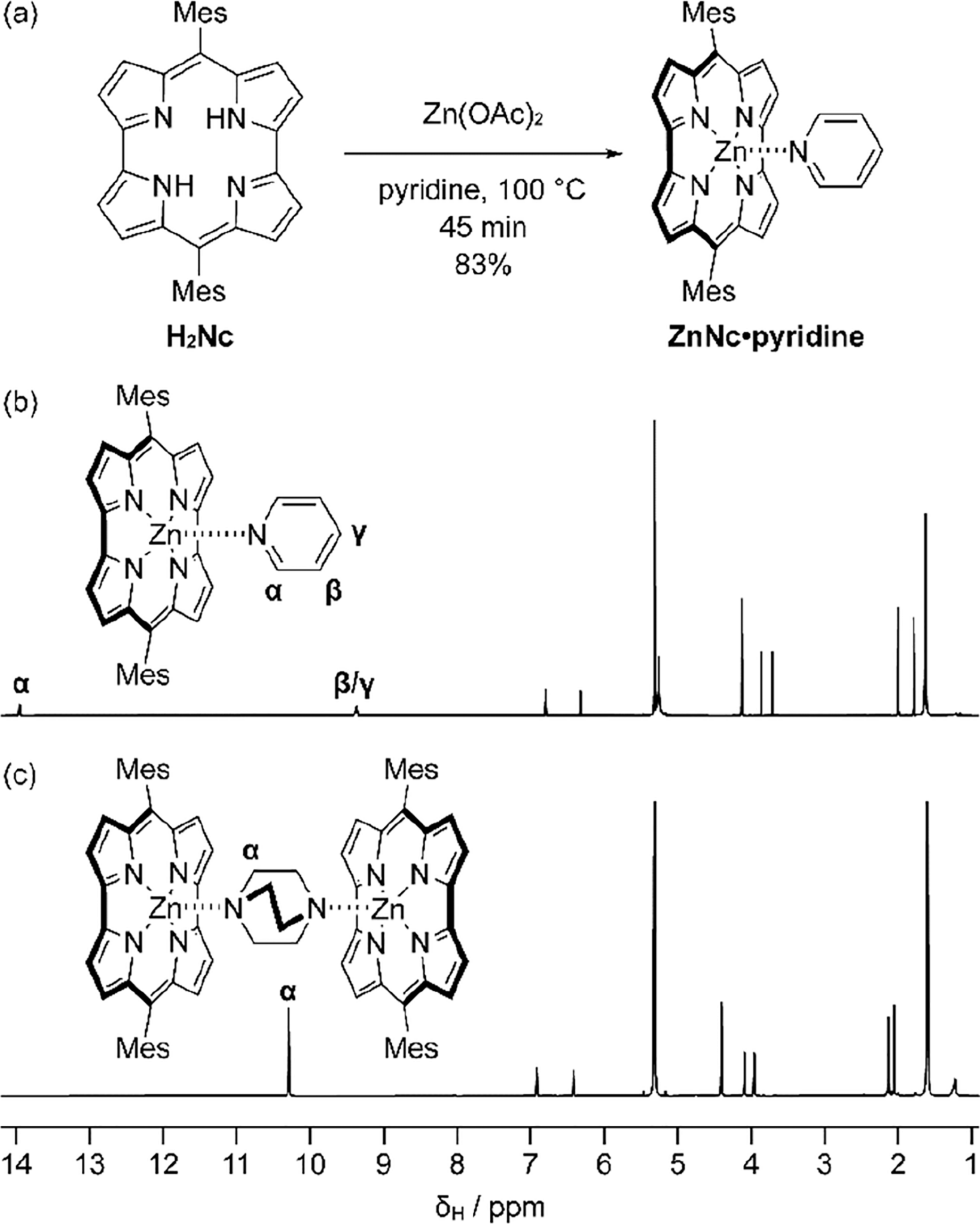
(a) Synthesis of ZnNc·pyridine. ^1^H NMR spectra (600 MHz, CD_2_Cl_2_) of (b) ZnNc·pyridine at 253 K, (c) (ZnNc)_2_·DABCO at 268 K. Mes is 2,4,6-trimethylphenyl (mesityl).

Attempts to purify the ZnNc·pyridine complex by column chromatography (SiO_2_ and Al_2_O_3_) resulted in demetalation to H_2_Nc. Recrystallization of the pyridine reaction solution with methanol afforded the pure ZnNc·pyridine complex as brown crystals. ^1^H NMR analysis of these crystals clearly reveals the deshielded bound pyridine signals at 13.95 and 9.38 ppm (Figure 1b). We assign the signal at 13.95 ppm to the α-pyridine protons (α), which are heavily deshielded due to their proximity to the antiaromatic norcorrole fragment. This assignment is corroborated by NOE signals between the pyridine alpha protons and the ZnNc mesityl CH_3_ protons (SI Figure S6). The ZnNc·pyridine complex is stable as a solid but slowly hydrolyses to H_2_Nc in the presence of water in solution.

Figure 2 shows the UV–visible absorption spectrum of the ZnNc·pyridine complex in toluene. ZnNc has a λ_max_ of 496 nm, which is red-shifted compared to NiNc, PdNc, and H_2_Nc (SI Table S1).^6,17^ Voltammetry reveals that the electrochemical bandgap of ZnNc·pyridine (1.16 V) is intermediate between those of H_2_Nc (1.38 V) and the reported gap of NiNc (1.08 V).^6^ For full details, see SI section S7. The redox processes occur at much lower potentials in ZnNc: whereas the first reduction of ZnNc·pyridine occurs at −1.44 V (vs Fc/Fc^+^), the first reduction of NiNc occurs at −0.92 V.^6^ These differences might be attributed to the curvature of ZnNc·pyridine resulting in weaker π-orbital overlap and reducing the degree to which ZnNc suffers antiaromatic instability. For comparison, the first reduction of the aromatic Ni(II) oxacorrole occurs at −1.75 V.^6^

**Figure 2:**
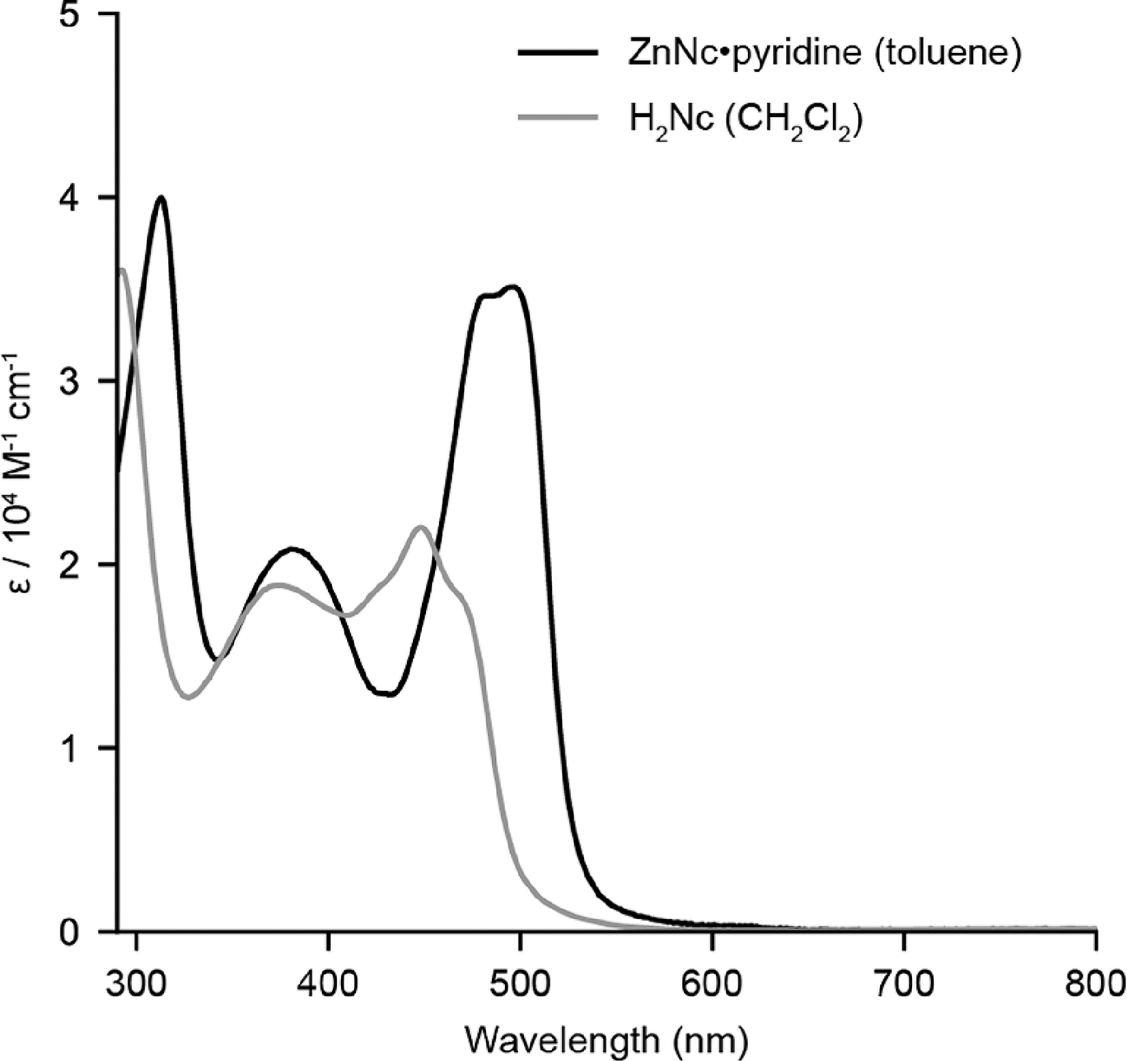
UV–visible absorption spectrum of ZnNc·pyridine in toluene and H_2_Nc in CH_2_Cl_2_. The extinction coefficients for H_2_Nc are from the literature.^17^

X-ray crystallography of ZnNc·pyridine reveals a distinctly bowl-shaped structure, in which the Zn^2+^ sits approximately 1.88 Å above the plane described by the eight pyrrole carbon atoms (see SI section S6, Figure S16 for more crystallographic information). Geometry and ^1^H NMR calculations using density functional theory (DFT) at the BLYP35/6-311G* level (SI section S8) further support our assignment of a bowl-shaped structure and reproduce the asymmetry observed in the experimental ^1^H NMR spectra. Nucleus independent chemical shifts (NICS)^24^ reveal higher deshielding on the concave face of the ZnNc complex, which explains the significantly stronger deshielding of *o*-CH_3_ and Ar–H protons (SI Figure S20). Anisotropy of the induced current density (AICD)^25^ calculations reveal the paratropic ring currents on the convex and concave faces of ZnNc·pyridine (SI Figure S19). The paratropic ring current traces the shortest 16 π-electron pathway.

Using NMR, we followed the displacement of pyridine from the ZnNc·pyridine complex with the bidentate ligand DABCO. DABCO, which comprises two diametrically opposed sp^3^ nitrogen atoms, should be a stronger ligand than pyridine. After the initial addition of DABCO, a sharp new signal appears at 9.52 ppm in the ^1^H NMR spectrum (Figure 1c and Figure 3), as well as signals corresponding to β-protons of a new norcorrole species (SI section S5). We assign the signal at 9.52 ppm to the protons of DABCO in a symmetric 2:1 (ZnNc)_2_·DABCO complex. The protons of free DABCO resonate at 2.43 ppm in toluene-*d*_8_, while the protons of DABCO in (ZnNc)_2_·DABCO are shifted downfield by 7.09 ppm because of the additive deshielding effects from the two antiaromatic ZnNc fragments. As we increase the concentration of DABCO, the 1:1 ZnNc·DABCO complex becomes the dominant species as evidenced by the triplet signals at 7.69 and 4.05 ppm, which are now magnetically inequivalent because ZnNc is only bound to one side of the DABCO ligand (SI section S5). We isolated (ZnNc)_2_·DABCO by carefully adding 0.5 equiv of DABCO to ZnNc·pyridine, then repeatedly adding and evaporating toluene to remove the liberated pyridine. The titration data required careful treatment using both curve-fitting and Bayesian methods, since we were unable to observe free ZnNc (see SI section S5). We found that the binding constant of ZnNc to DABCO is approximately 10^9^ M^–1^; the 1:2 complex has a formation constant of 10^17^ M^–2^, and the binding constant of ZnNc to pyridine is approximately 10^7^ M^–1^. A full error analysis is available in the Supporting Information. The 1:1 binding constants are approximately 10^3^ times higher (∼17 kJ/mol) than analogous binding constants for zinc porphyrin,^26^ likely reflecting that the norcorrole ligand’s curvature exposes the Zn^2+^ for axial binding. The deshielding of the NMR resonances on the DABCO or pyridine ligands is consistent with a ring current susceptibility of ∼35–40 nA/T in zinc norcorrole (see SI section S9), which is very similar in magnitude (albeit of opposite sign) to that reported for zinc porphyrins.^27^

**Figure 3:**
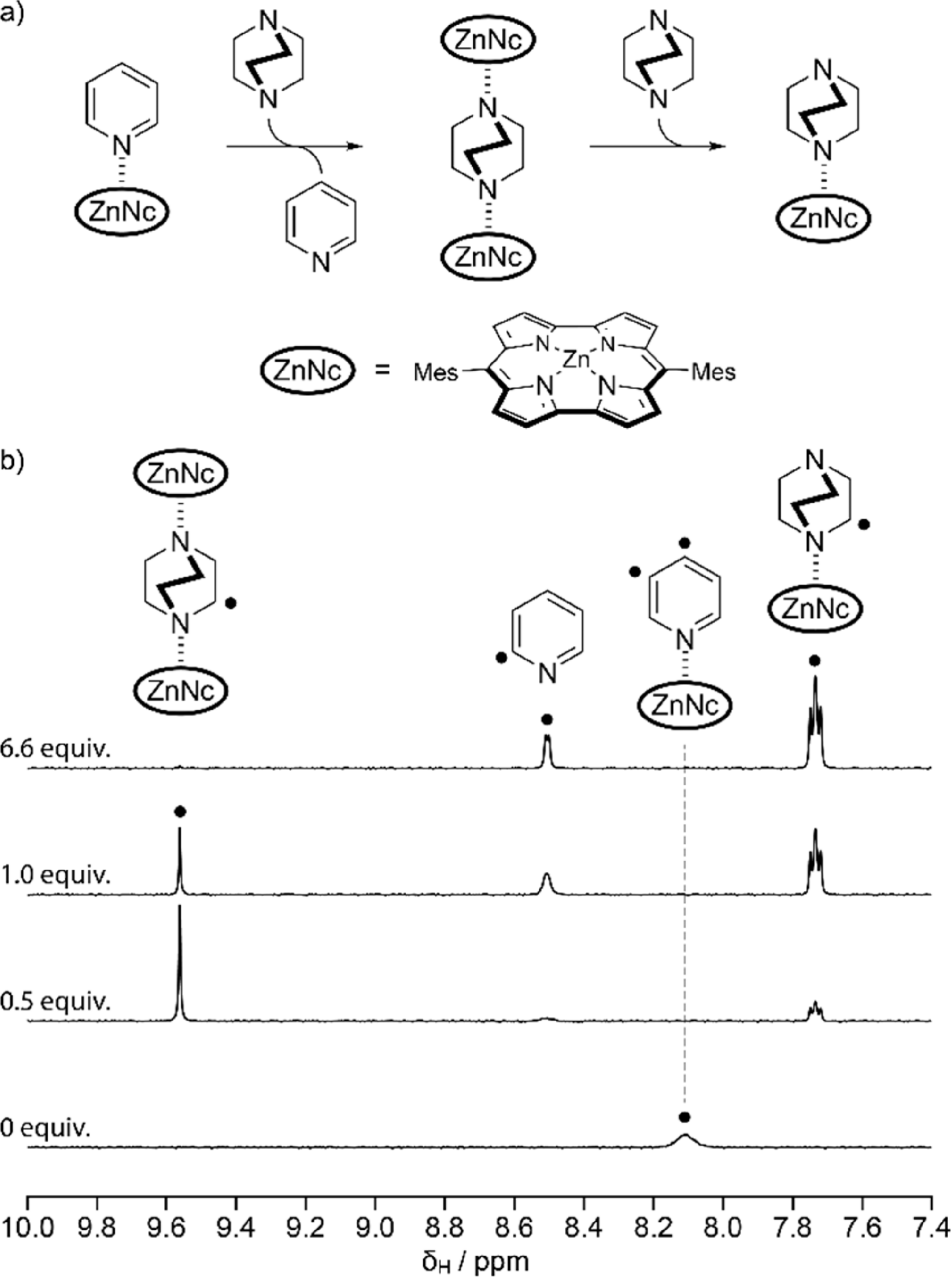
Titration of DABCO into a solution of ZnNc·pyridine (a) reaction scheme for the ligand displacement reactions; (b) ^1^H NMR spectra of ZnNc·pyridine in toluene-*d*_8_ (500 MHz, 298 K) with different molar ratios of DABCO. Black spots indicate corresponding proton assignments and signals. Mes = 2,4,6-trimethylphenyl.

Single crystals of (ZnNc)_2_·DABCO were grown by vapor diffusion of diethyl ether into a concentrated solution of the complex in toluene. The structure crystallized in the triclinic space group *P*1̅ with one complete (ZnNc)_2_·DABCO and two ether solvents of crystallization present in the asymmetric unit. The diffraction data reveal a bend about the DABCO ligand, such that when a plane is fitted between the four nitrogen atoms of each norcorrole fragment, the fold angle between these planes is 19.4°. The norcorroles of each dimer are also twisted 18° with respect to each other (Figure 4). The bowl depths of the ZnNc fragments, calculated as the distance between Zn^2+^ and a least-squares plane of the eight constituent β-pyrrole carbons, are 1.77 and 1.83 Å, meaning Zn^2+^ sits further out of the norcorrole plane in the axially ligated (ZnNc)_2_·DABCO complex than Pd^2+^ does in PdNc (1.21 Å).^17^ We can compare the curvature of simple convex molecular fragments such as these by fitting their atomic coordinates to the surface of a sphere. A smaller radius is associated with higher curvature. Fitting the heavy atoms only (excluding metals so as to reduce the effect of the axial ligands), we find that the norcorrole fragments in ZnNc complexes are more curved (*r* = 5.6 and 5.8 Å for pyridine and DABCO complexes, respectively) than PdNc (6.5 Å). The coordinates of corannulene^28^ fit to a sphere of radius 5.9 Å, providing a useful benchmark for the curvature of the π-system in ZnNc. Crystallographic data for the pyridine and DABCO complexes of ZnNc have been deposited to Cambridge Crystallographic Data Centre (numbers 2340208 and 2334079).

**Figure 4:**
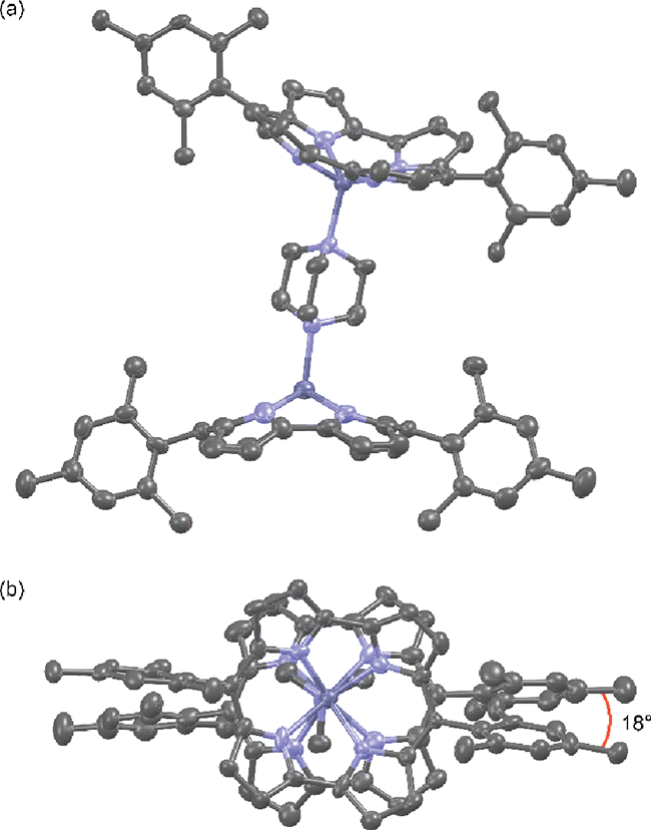
X-ray crystal structure of (ZnNc)_2_·DABCO (a) side view, (b) top view. Thermal ellipsoids are set to 50% probability. Hydrogen atoms are removed for clarity. Diethyl ether solvent molecules (not shown) sit in the concave face of each zinc norcorrole.

In conclusion, we report zinc norcorrole complexes as bowl-shaped molecules, evidenced by crystallography and NMR spectroscopy. We do not observe bowl inversion on the NMR time scale. Zinc norcorrole can act as a ligand acceptor by coordinating pyridine. By a ligand-exchange protocol, the strong ligand DABCO has been bound to zinc norcorrole to generate a supramolecular sandwich structure. The ability of zinc norcorrole to coordinate axial ligands offers a promising method to generate more complex supramolecular compounds, thus allowing the exploration of antiaromaticity in large structures. The complexes explored here represent the first supramolecular complexes of norcorrole which involve coordinating a ligand to the central metal of the antiaromatic tetrapyrrole.

## Supporting Information Available

Synthetic procedures, NMR spectra, electrochemistry, computational chemistry and analysis, binding constant analysis, and crystallographic details (PDF)
