# Supplementary material for: Probing the Antiaromaticity and Coordination Chemistry of Bowl-Shaped Zinc(II) Norcorrole
Source: Inorg Chem. 2024 May 23;63(22):10103–7. doi: 10.1021/acs.inorgchem.4c01146 (PMC11152050; doi:10.1021/acs.inorgchem.4c01146)
Supplement: Supplementary file 1 — ic4c01146_si_001.pdf [file ic4c01146_si_001.pdf]

Supporting Information

Probing the Antiaromaticity and Coordination Chemistry of Bowl-Shaped Zinc(II) Norcorrole

David Bradley,[a] Ruoming Tian,[b] Mohan M. Bhadbhade,[b] Lauren K. Macreadie,[a] Chowdhury Hasan Sarowar,[c] Martin D. Peeks*,[a]

[a] School of Chemistry, UNSW Sydney NSW 2052 Australia

[b] Mark Wainwright Analytical Centre, UNSW Sydney NSW 2052 Australia

[c] Bioanalytical Mass Spectrometry Facility, Mark Wainwright Analytical Centre, UNSW Sydney, NSW 2052 Australia

* Corresponding author: m.peeks@unsw.edu.au

Contents

S1. General methods 2

S2. Synthetic procedures 3

Synthesis of the ZnNc•pyridine complex 3

Synthesis of $(ZnNc)_2$•DABCO 4

S3. NMR Spectra 5

S4. UV-visible absorption spectrum 10

S5.Analysis of binding constants 11

S6. X-Ray diffraction analysis 17

S7. Electrochemistry 20

S8. Theoretical calculations 21

S9. Ring current analysis 24

S10. References 24

S1. General methods

NMR spectra were recorded on a 600 MHz Bruker Avance III HD with a TCI helium probe, 500 MHz Bruker Avance III with a TBI probe, or a 400 MHz Bruker Avance III fitted with a Prodigy cryoprobe. Chemical shifts are reported relative to that of the protonated solvent: $CHDCl_2$ (for CD_2Cl_2) at δ 5.32 ppm or the protio-form of toluene-d_8, at 6.97 ppm. UV-visible absorption spectra were recorded on an Agilent Cary 60 spectrophotometer and UV-vis-NIR absorption spectra were recorded on a PerkinElmer LAMBDA 1050 spectrophotometer. Mass spectra were recorded on a Thermo LTQ Orbitrap XL. X-ray diffraction measurements were carried out at 120 K on a Bruker D8 Quest diffractometer with Mo-Kα radiation. Solution state cyclic voltammetry was performed using a PalmSens4 potentiostat with ferrocene as an internal reference.

Pyridine was distilled then dried over 4 Å molecular sieves. Unless otherwise mentioned, all other solvents and reagents were purchased from commercial suppliers and used without further purification.

S2. Synthetic procedures

Freebase norcorrole was synthesized according to a literature procedure.[1]

Synthesis of the ZnNc•pyridine complex

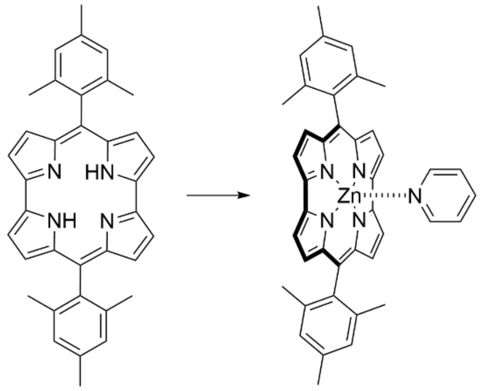

Dry pyridine (1.5 mL) was added to a mixture of freebase norcorrole (8.4 mg, 16.1 μmol) and $Zn(OAc)_2$ (34 mg, 0.19 mmol, 11.5 equiv.). The solution was stirred at 100 °C for 45 minutes then cooled to room temperature. MeOH (12 mL) was added to the reaction mixture and the resulting precipitate was collected by filtration and washed with MeOH and hexane, affording the ZnNc•pyridine complex as brown needles (8.9 mg, 13.4 μmol, 83%).

^{1}H NMR (600 MHz, 253 K, CD_2Cl_2) δ_H, ppm: 13.95 (d, J = 4.0 Hz, 2H), 9.40-9.35 (m, 3H), 6.79 (s, 2H), 6.32 (s, 2H), 4.13 (s, 6H), 3.86 (d, J = 4.0 Hz, 2H), 3.72 (d, J = 4.0 Hz, 2H), 2.00 (s, 6H), 1.79 (s, 6H). ^{13}C NMR (151 MHz, 253 K, CD_2Cl_2) δ_C, ppm: 156.00, 153.91, 146.20, 142.39, 139.59, 136.71, 136.06, 135.74, 132.16, 129.78, 128.60, 128.37, 127.95, 117.13, 20.64, 19.48, 16.80.

UV-vis (toluene): λ_{max} nm (ε [M^{-1} cm^{-1}]) 496 (35000), 382 (21000).

HRMS (ESI): m/z = 601.1935, calc'd for $[C_{36}H_{30}N_4Zn+H_2O+H]^+$ = 601.1940.

Synthesis of $(ZnNc)_2$•DABCO

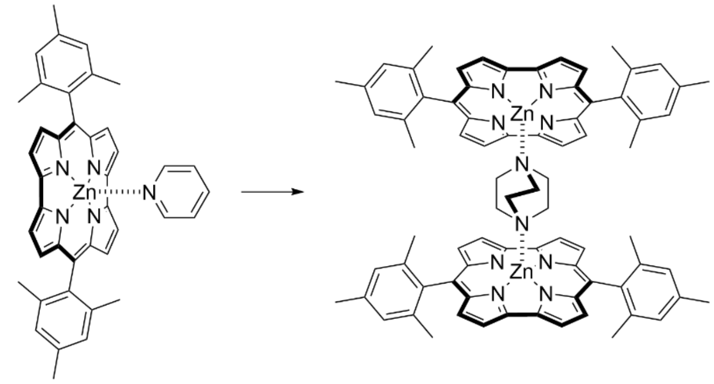

The concentration of a solution of the ZnNc•pyridine complex in toluene was determined by UV-visible absorption spectroscopy then the solvent was removed under vacuum. An example procedure follows based on 0.33 mg of ZnNc•pyridine.

A 2.6 mM stock solution of 4-diazabicyclo[2.2.2]octane (29.3 mg, 0.26 mmol) in toluene (100 mL) was prepared. An aliquot of a 2.6 mM DABCO stock solution in toluene (0.11 mL, 0.29 µmol, 0.5 eq. of DABCO) was added to the ZnNc•pyridine (0.57 µmol, 0.33 mg, determined by UV-vis spectroscopy) then the solvents were removed under vacuum. Toluene (20 mL) was added to the brown residue and then removed by evaporation. This dissolution-evaporation process was repeated five times to ensure complete removal of pyridine. The brown residue was then washed with a small amount of hexane and dried in vacuo, affording $(ZnNc)_2$•DABCO as a brown solid in quantitative yield by NMR.

^{1}H NMR (600 MHz, 268 K, CD_2Cl_2) δ_H, ppm: 10.29 (s, 12H), 6.92 (s, 4H), 6.42 (s, 4H), 4.41 (s, 12H), 4.09 (d, J = 4.2 Hz, 8H), 3.96 (d, J = 4.2 Hz, 8H), 2.13 (s, 12H), 2.06 (s, 12H). ^{13}C NMR (101 MHz, 298 K, CD_2Cl_2) δ_C, ppm: 156.65, 147.11, 140.59, 137.25, 136.86, 136.73, 132.64, 130.41, 128.93, 128.54, 118.08, 55.15, 20.92, 20.14, 17.60.

S3. NMR Spectra

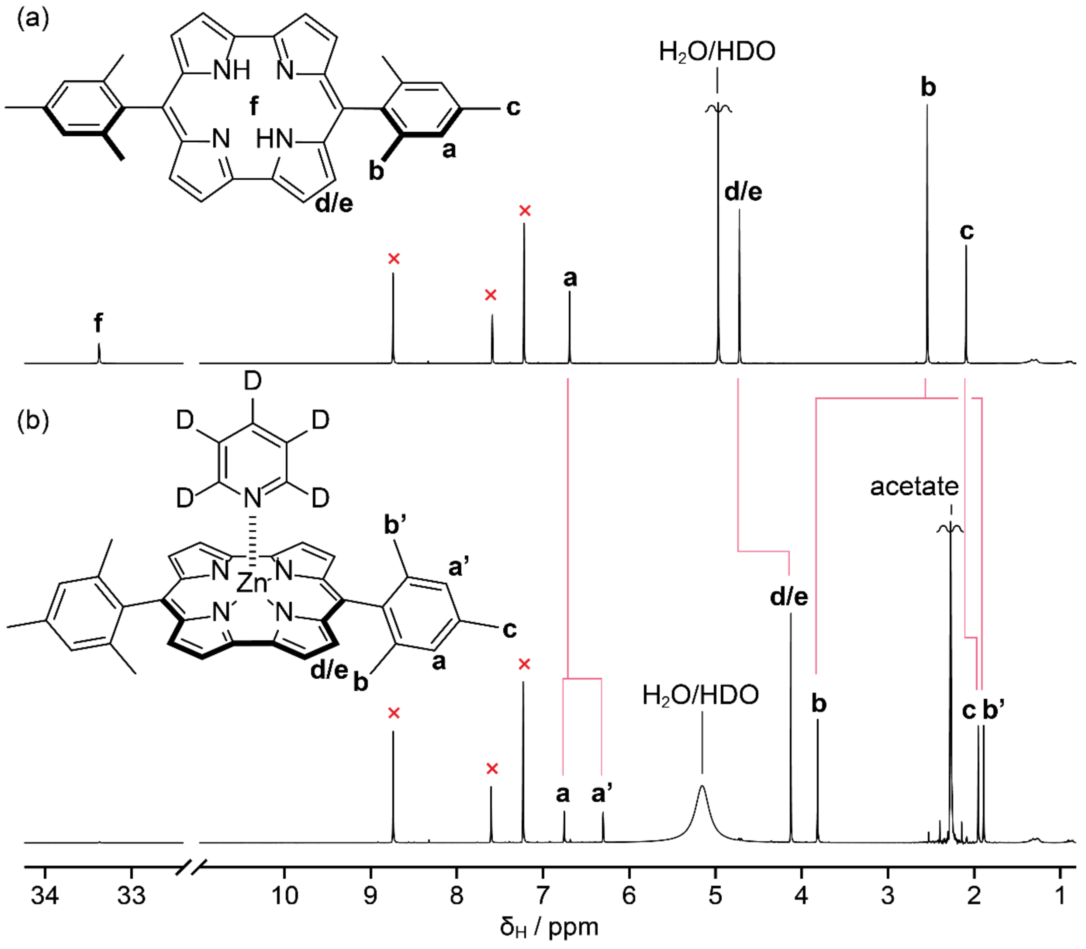

Figure S1 ^1H NMR (500 MHz, 298 K, pyridine-d_5) spectrum of (a) freebase norcorrole and (b) ZnNc•pyridine-d_5 after addition of $Zn(OAc)_2$ and stirring at 100 °C for 40 minutes. These spectra show the desymmetrization of the norcorrole core in zinc norcorrole. Red crosses denote peaks which arise from residual protio-pyridine.

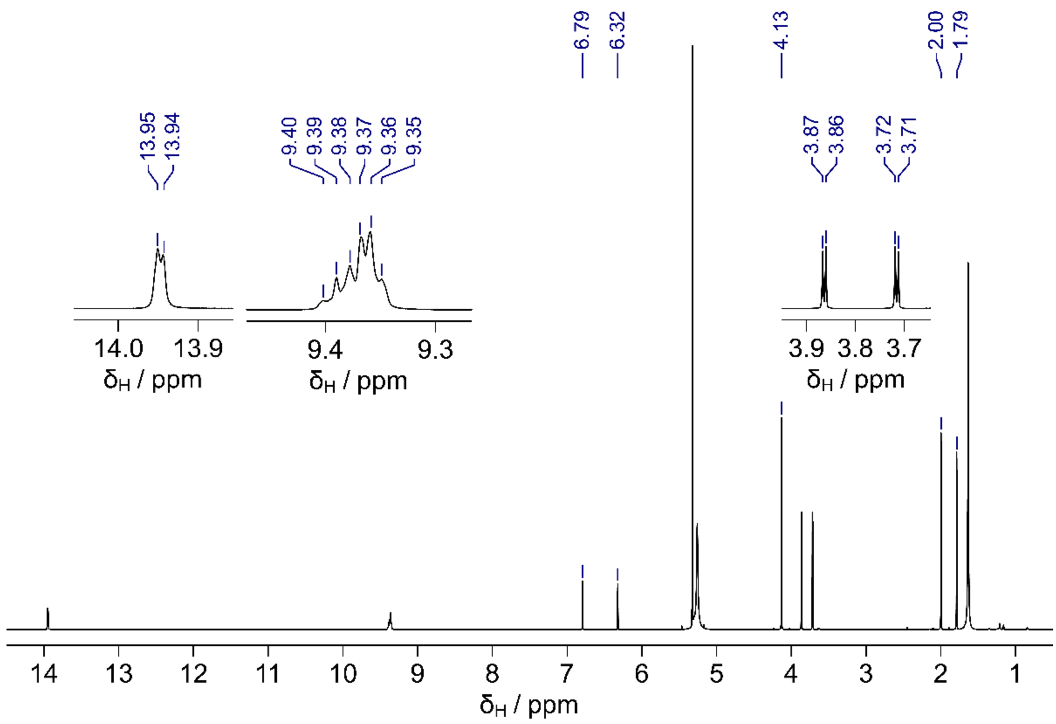

Figure S2 ^{1}H NMR (600 MHz, 253 K, CD_2Cl_2) spectrum of ZnNc•pyridine.

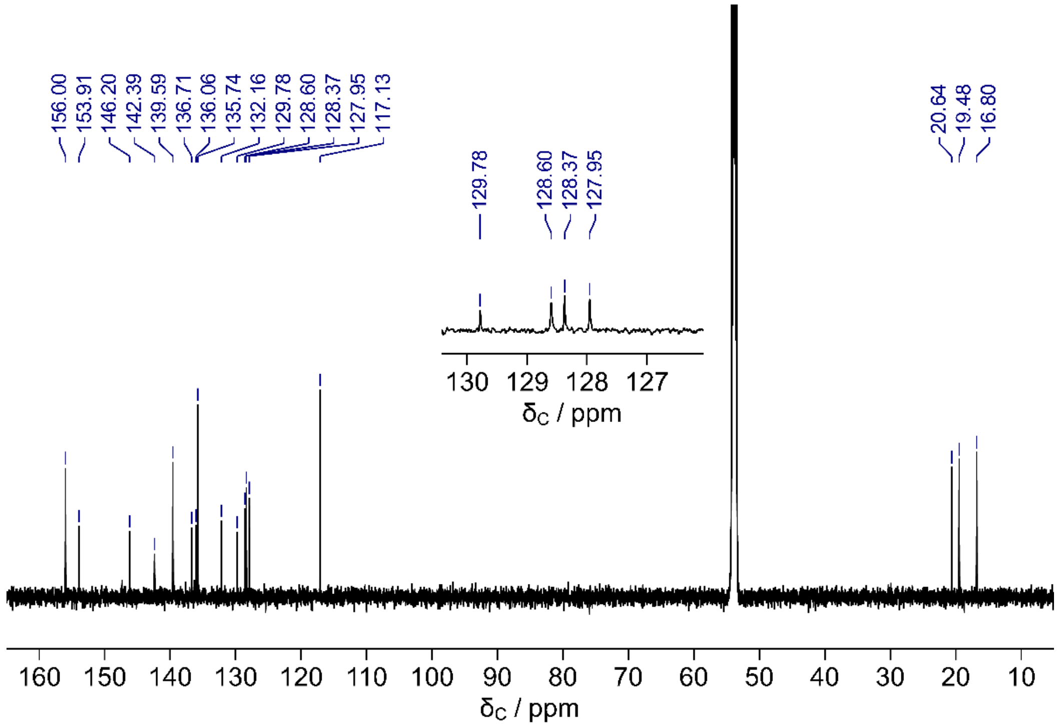

Figure S3 ^{13}C NMR (151 MHz, 253 K, CD_2Cl_2) spectrum of ZnNc•pyridine complex.

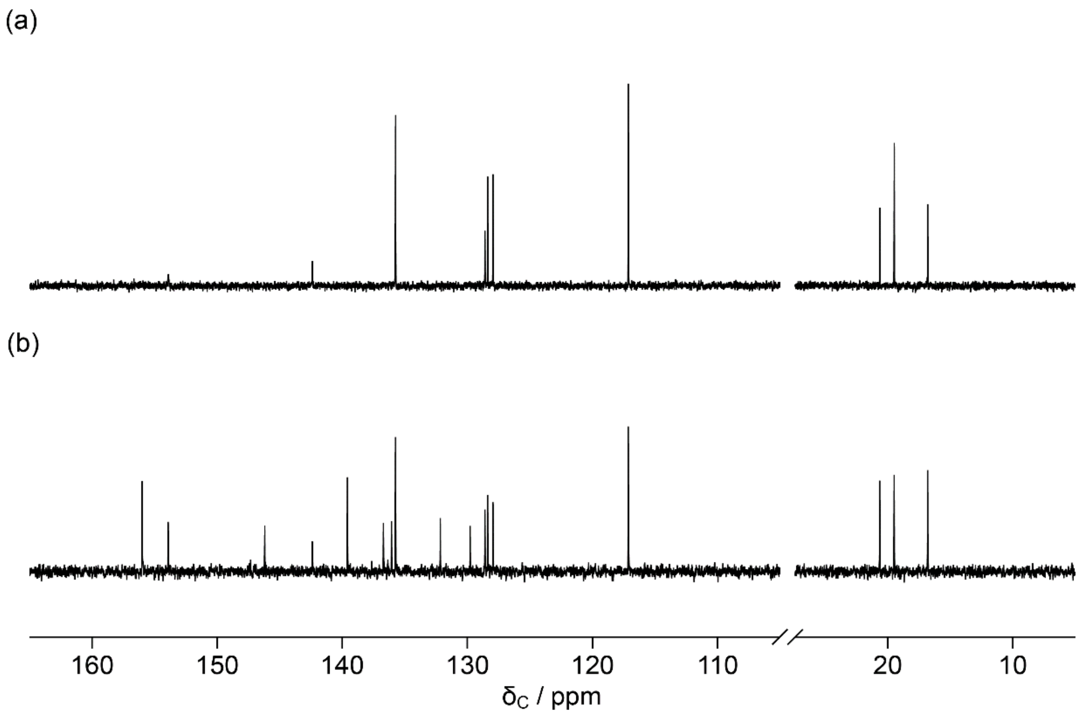

Figure S4 (a) DEPT 135 and (b) ^{13}C (151 MHz, 253 K, CD_2Cl_2) spectra of ZnNc•pyridine.

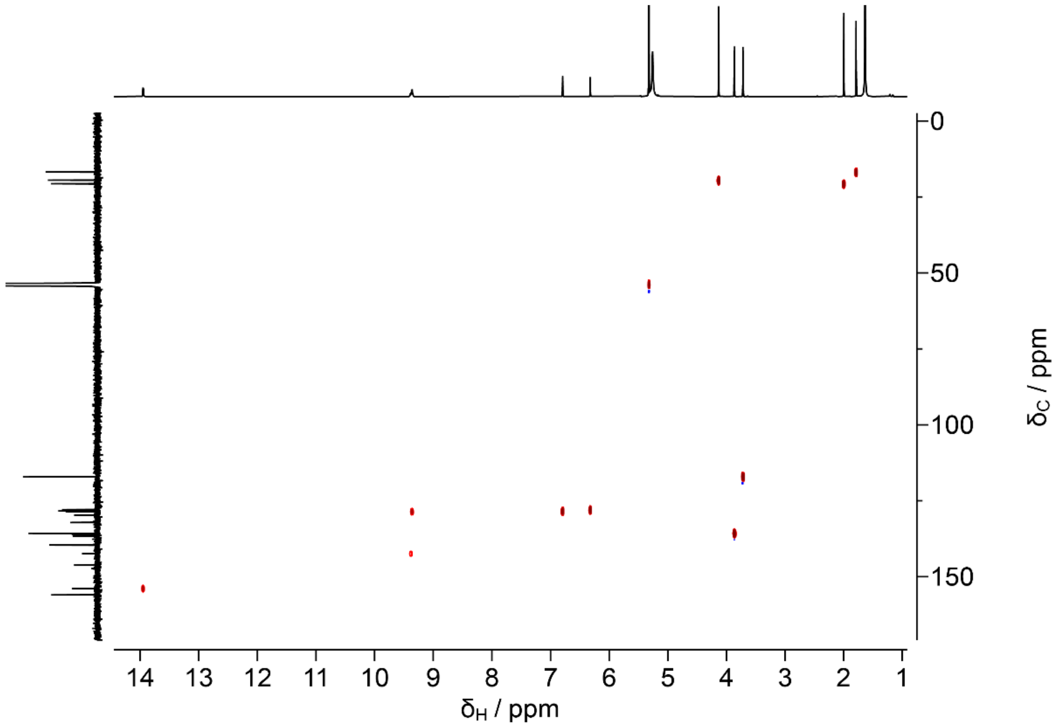

Figure S5 ^{1}H – ^{13}C HSQC (600 MHz, 253 K, CD_2Cl_2) spectrum of ZnNc•pyridine.

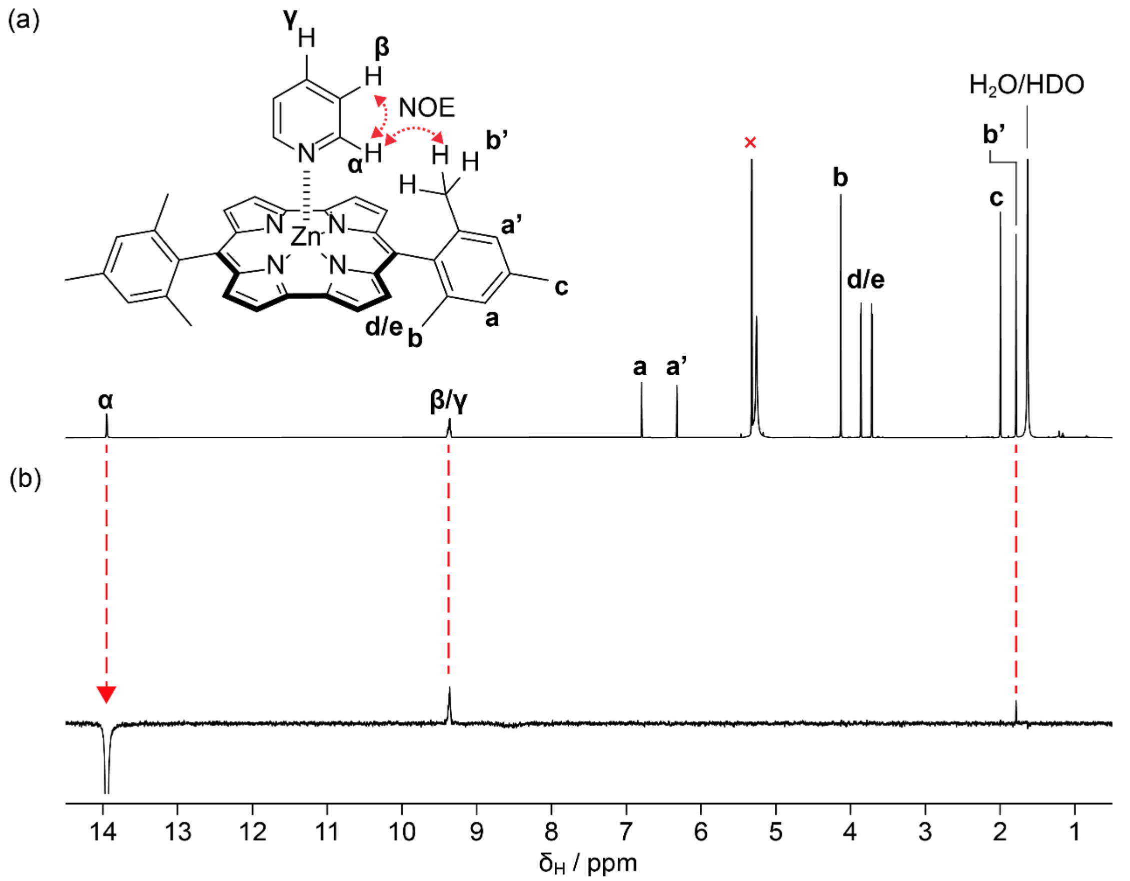

Figure S6 (a) ^{1}H NMR and (b) 1D NOESY (mixing time = 0.5 seconds, 600 MHz, 253 K, CD_2Cl_2) spectra of ZnNc•pyridine. The red cross indicates a residual CH_2Cl_2 solvent peak.

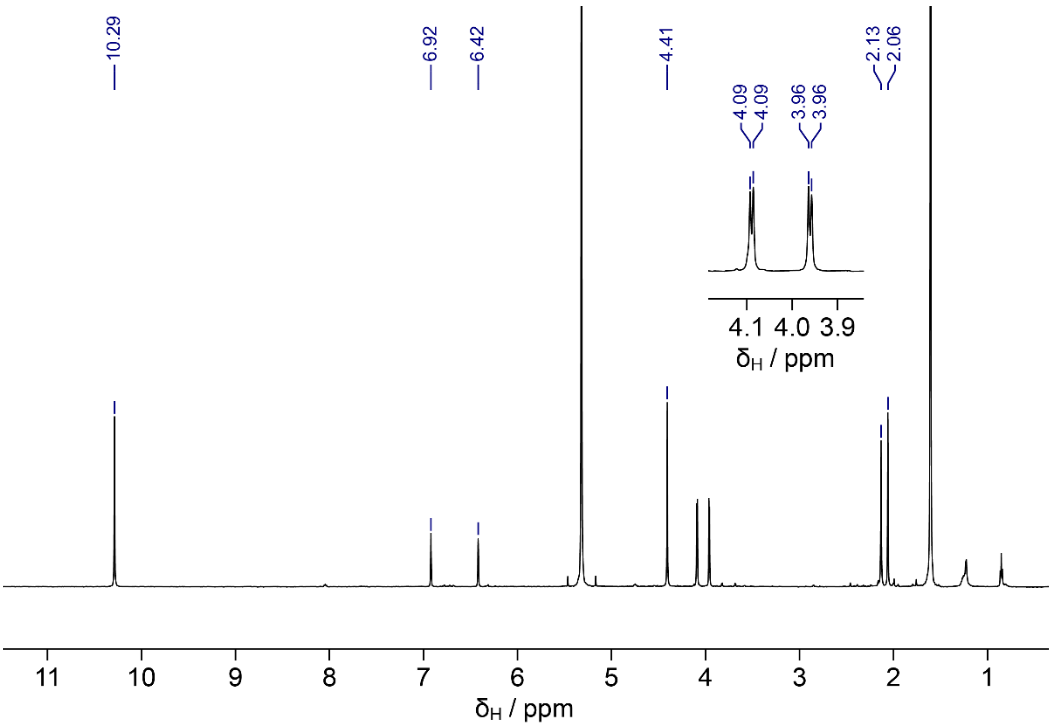

Figure S7 ^{1}H NMR (600 MHz, 268 K, CD_2Cl_2) spectrum of $(ZnNc)_2$•DABCO.

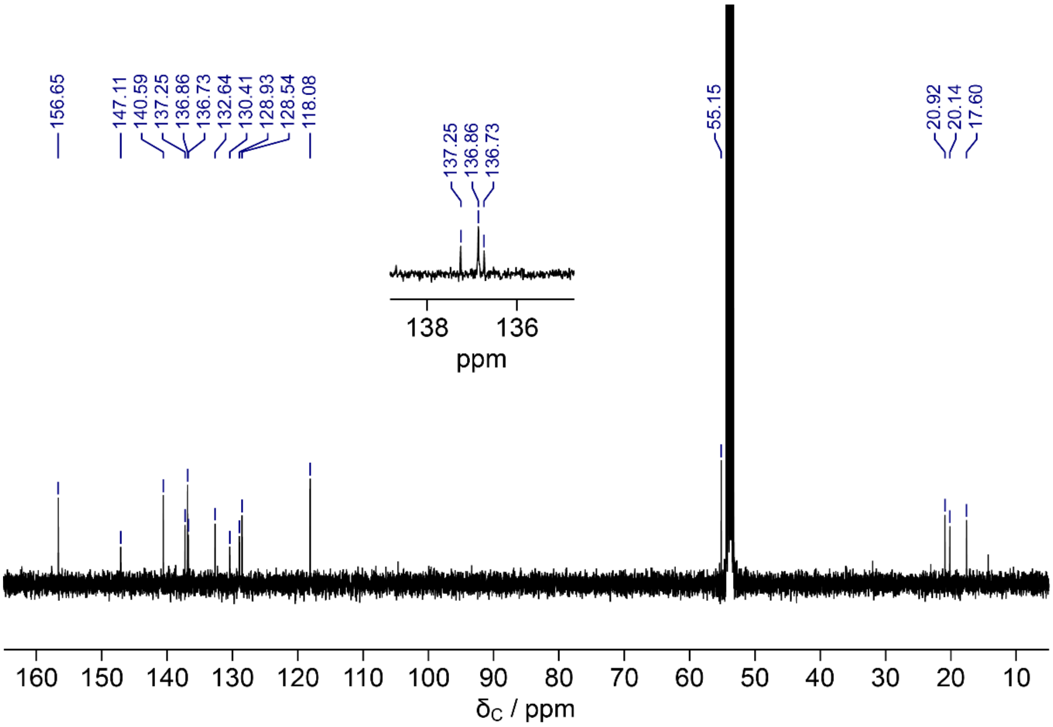

Figure S8 ^{13}C NMR (101 MHz, 298 K, CD_2Cl_2) spectrum of $(ZnNc)_2$•DABCO.

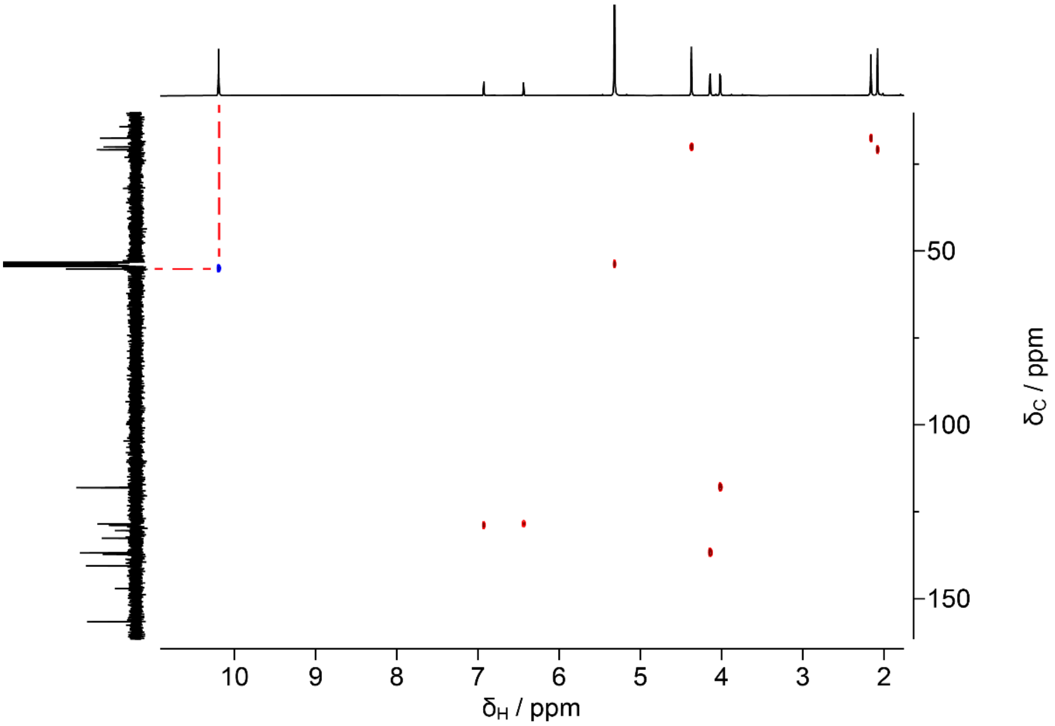

Figure S9 ^{1}H – ^{13}C HSQC (600 MHz, 151 MHz, 298 K, CD_2Cl_2) spectrum of $(ZnNc)_2$•DABCO.

S4. UV-visible absorption spectrum

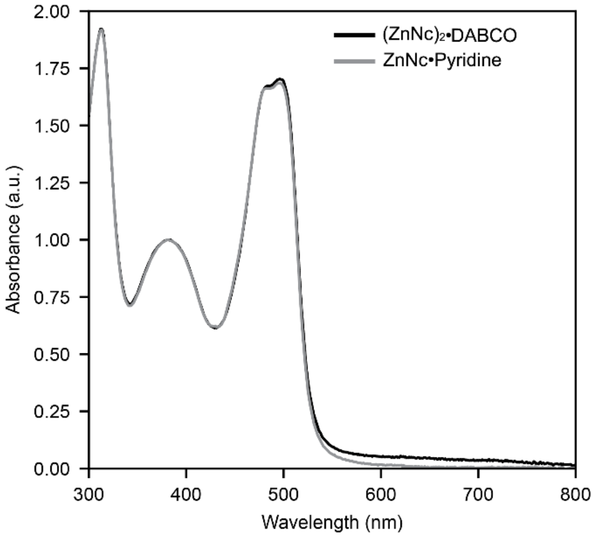

Figure S10 UV-visible absorption spectrum of $(ZnNc)_2$•DABCO and ZnNc•pyridine in toluene.

Table S1 UV-visible absorption data for selected mesityl metallonorcorroles.

Compound	λ_{max} / nm	ε / M^{-1} cm^{-1}	Solvent	Reference
ZnNc•Pyridine	496	35000	Toluene	This work
H_2Nc	449	22000	CH_2Cl_2	1
NiNc	431	46000	CH_2Cl_2	2
CuNc	471	28000	CH_2Cl_2	1
PdNc	414	30000	CH_2Cl_2	1

S5.Analysis of binding constants

Free ZnNc could not be prepared, and so the titration involved addition of DABCO (as a stock solution of DABCO and ZnNc•pyridine in toluene-d8) to a solution of ZnNc•pyridine (0.2 mM) in toluene-d8. A constant ZnNc concentration of ~0.2 mM was maintained throughout the titration.

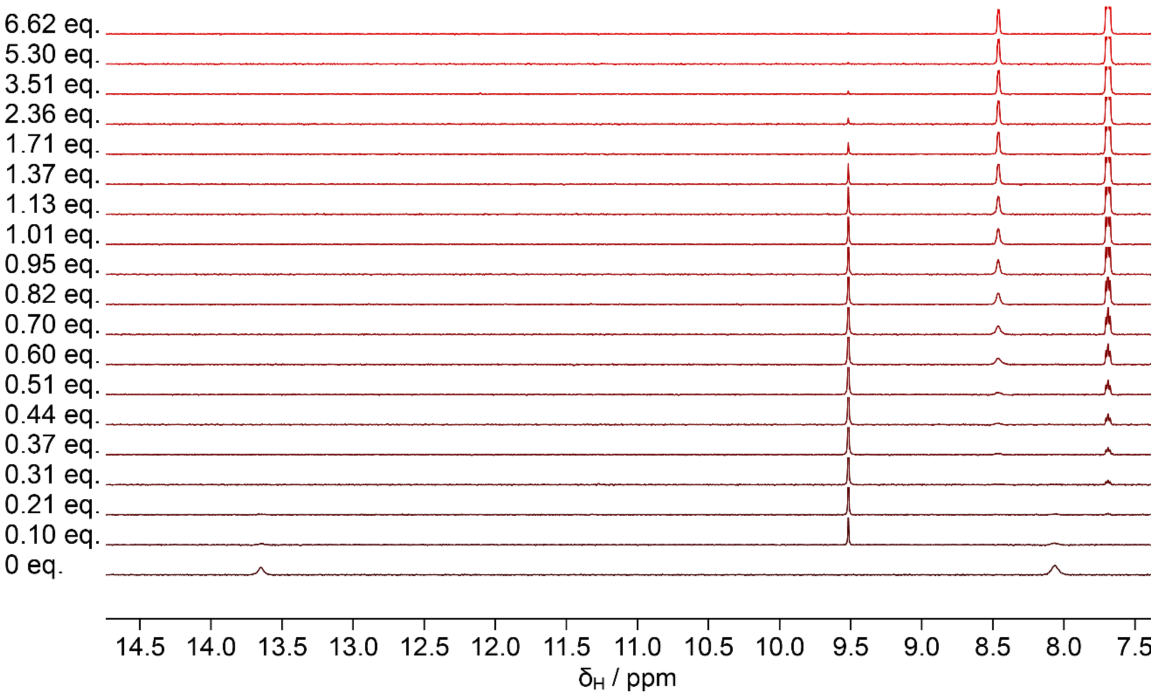

Figure S11 ^{1}H NMR (500 MHz, 298 K, toluene-d8) spectra of the titration of DABCO into ZnNc•pyridine showing the bound-ligand region. Numbers on the left denote the equivalents of DABCO added to the sample.

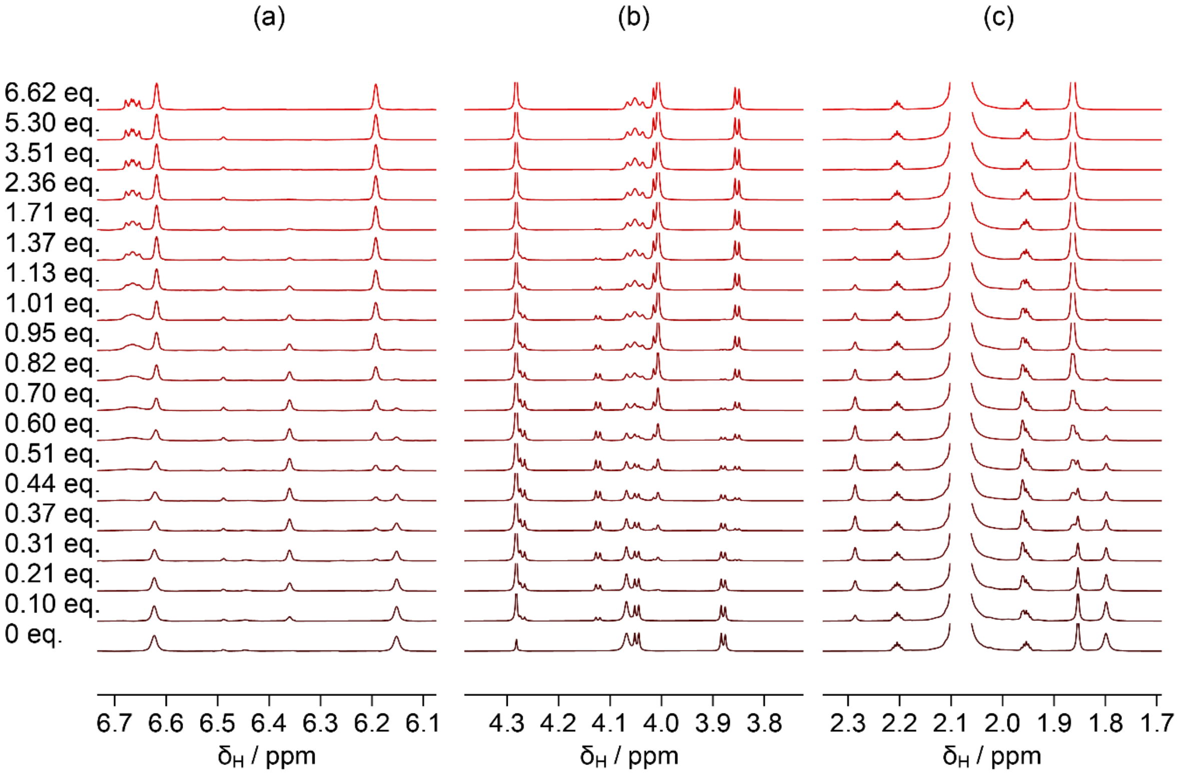

Figure S12 ^{1}H NMR (500 MHz, 298 K, toluene-d_8) spectra of the titration of DABCO into ZnNc•pyridine showing the (a) ArH, (b) β, and (c) CH_3 regions. Numbers on the left denote the equivalents of DABCO added to the sample.

Table S2 Species concentrations and ^{1}H NMR integrals from the titration of DABCO into ZnNc•pyridine. These integrals correspond to β-protons of ZnNc•pyridine, ZnNc•DABCO, and $(ZnNc)_2$•DABCO at 3.88 ppm, 3.85 ppm, and 4.12 ppm respectively. The integrals of the β-protons of $(ZnNc)_2$•DABCO were halved because they correspond to 8 protons, whereas the analogous signal in the other two species corresponds to 4 protons.

Species concentration		Equivalents	Species integral		
[ZnNc]/mM	[DABCO]/mM	[DABCO]/[ZnNc]	ZnNc•pyridine	ZnNc•DABCO	$(ZnNc)_2$•DABCO
0.199	0.021	0.10	9597	68	38
0.199	0.041	0.21	7755	113	973
0.199	0.061	0.31	6086	315	1643
0.199	0.074	0.37	4628	719	2141
0.199	0.087	0.44	3781	1117	2373
0.199	0.100	0.51	2960	1566	2460
0.199	0.120	0.60	2345	2116	2523
0.199	0.139	0.70	1734	3174	2353
0.199	0.164	0.82	1222	4047	2097
0.199	0.188	0.95	792	5425	1733
0.199	0.201	1.01	596	6698	1281
0.199	0.225	1.13	494	7183	1102
0.199	0.272	1.37	361	7902	839
0.199	0.339	1.71	253	8915	429
0.199	0.470	2.36	126	9377	273
0.199	0.698	3.51	122	9784	179
0.199	1.053	5.30	124	9974	129
0.199	1.317	6.62	129	10079	70

The system can be described by three speciation constants:

$$\mathrm{ZnNc + pyridine \leftrightharpoons ZnNc \cdot pyridine} \qquad \beta_1$$

$$\mathrm{ZnNc + DABCO \leftrightharpoons ZnNc \cdot DABCO} \qquad \beta_2$$

$$\mathrm{2\,ZnNc + DABCO \leftrightharpoons ZnNc_2 \cdot DABCO} \qquad \beta_3$$

The binding problem is not straightforward to solve because we are unable to observe free ZnNc. We used a non-linear optimization routine to fit the values of the speciation constants (β, above). For each guess vector of equilibrium constants, the speciation problem was solved using the Newton-Raphson method following ref [3] and the results compared to the experimentally-observed concentrations from the NMR integrals in Table S2. All of the code describing the fitting process is available on github (https://github.com/martinp23/znnc-fitting) and is archived on Zenodo (tbc).

This model relies on the equilibrium processes being kinetically slow on the NMR timescale (*i.e.* in slow exchange). The pyridine resonances in ZnNc•pyr are broad (see, for example, Figure S11), and sharpen on cooling (Figure S2), which we attribute to restricted rotation of the pyridine group. In contrast, the norcorrole pyrrolic β-resonances of each species do not move and remain distinct during the titration, consistent with slow ligand exchange.

We began by fitting our model to the experimental data using the `least_squares` method from `scipy.optimize`, implemented through `lmfit`.[4,5] The results were strongly dependent on the initial guess conditions and choice of solver, suggesting that the fitting surface was relatively flat and prone to local minima. The speciation constants (β) were strongly correlated to each other. We used the best

results (*i.e.* those with the lowest sum-of-squares error) from the least squares fitting to further explore the fitting space using the Markov-Chain Monte Carlo method as implemented in the `emcee` package.[6] We chose to use this method because it allows the incorporation of an unknown experimental error (treated as normally distributed), and the application of chemically-reasonable priors (Table S3).

Table S3: Priors for Bayesian analysis of the binding problem.

Prior	Justification
$\log_{10}\beta_1 < \log_{10}\beta_2 < \log_{10}\beta_3$	This prior follows from the experimental data: DABCO (β_2) displaces pyridine (β_1) and so the former's speciation constant must be higher. The speciation constant for a 2:1 complex of ZnNc and DABCO (β_3) will be higher than that for the 1:1 complex (β_2). This prior is included to prevent the Monte-Carlo system from exploring unfeasible solution space.
$3 < \log_{10}\beta_1 < 12$	The speciation constant of ZnNc•pyr must be much higher than 10^3 M^{-1} otherwise we would expect to see free ZnNc in solution at NMR concentrations (10^{-4} M). The upper bound sets a limit on what is likely to be a "chemically reasonable" speciation constant.
$3 < \log_{10}\beta_2 < 20$	As above.
$8 < \log_{10}\beta_3 < 30$	As above, except that the limiting speciation constants are scaled to reflect the 2:1 character of the binding.
Errors on experimentally-measured concentrations are normally distributed	Reasonable assumption, relies on central limit theorem.

We initialised 50 walkers by sampling log-normal distributions (variance = 0.1) about the least squares solution, and evolved the walkers over 20000 steps. This process took about 45 minutes of computer time on a standard desktop, using 8 cores, and benefitting from just-in-time optimization using `numba`. The autocorrelation time was approximately 68 steps, so we discarded 6800 steps and thinned every 100 steps. The corner plot in Figure S13 shows the results of the Markov-Chain Monte Carlo simulation, and lists the maximum likelihood estimates and 1σ error ranges, plotted using the `corner` package.[7] It is clear that the parameters $\log\beta_1$, $\log\beta_2$ and $\log\beta_3$ are very strongly correlated to one-another, but neither is correlated to the noise parameter ($\ln\sigma = -13; \sigma \approx 2 \times 10^{-6}$M). This value for the noise parameter is approximately consistent with the error in the NMR integration.

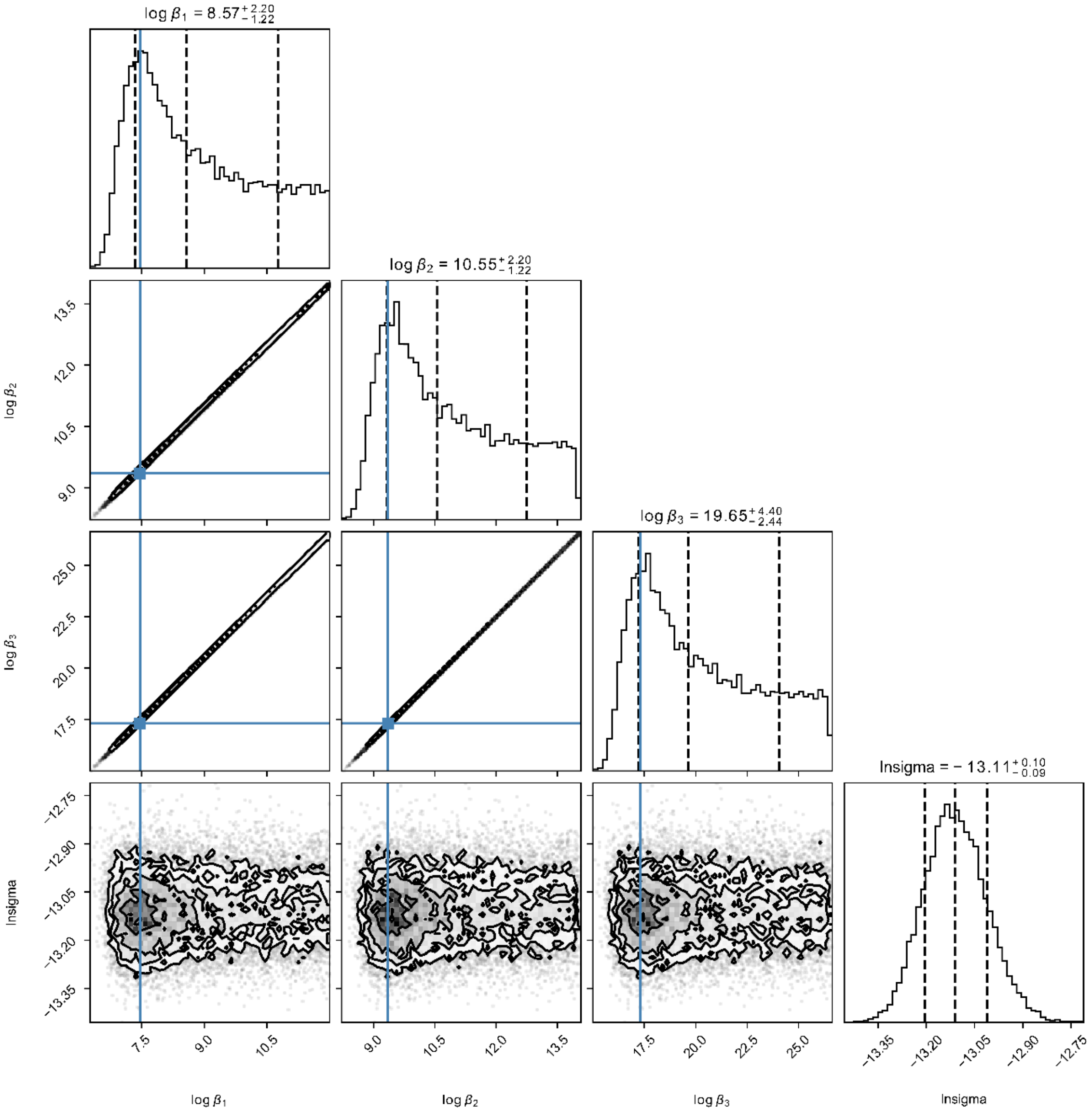

Figure S13 Corner plot showing the results from the Markov-Chain Monte-Carlo analysis of the NMR titration data from Table S2. The blue points/lines denote the solutions from the least-squares analysis (which excluded the error term "lnsigma"). The dotted lines denote the maximum-likelihood estimate and 1σ either side.

Due to the strong correlation between the fitted parameters, we explored whether the fitting problem could be simplified and so performed simulations varying only $\log_{10}\beta_1$, subject to:

$$\log_{10}\beta_2 = \log_{10}\beta_1 + 1.98$$

$$\log_{10}\beta_3 = 2\log_{10}\beta_1 + 2.51$$

For $\log_{10}\beta_1 < 6$, the simulation is unable to reproduce the experimental results because it underestimates the initial [ZnNc•pyr] (Figure S14e,f). However, for higher values of $\log_{10}\beta_1$ the simulation qualitatively matches the experimental data, even as $\log_{10}\beta_1$ approaches unrealistic values (*e.g.* $\log_{10}\beta_1 = 20$). A plot of sum-square-error versus $\log_{10}\beta_1$ reveals a shallow minimum at $\log_{10}\beta_1 = 7.4$ (Figure S15), which is consistent with the modal solution in the Monte-Carlo simulation.

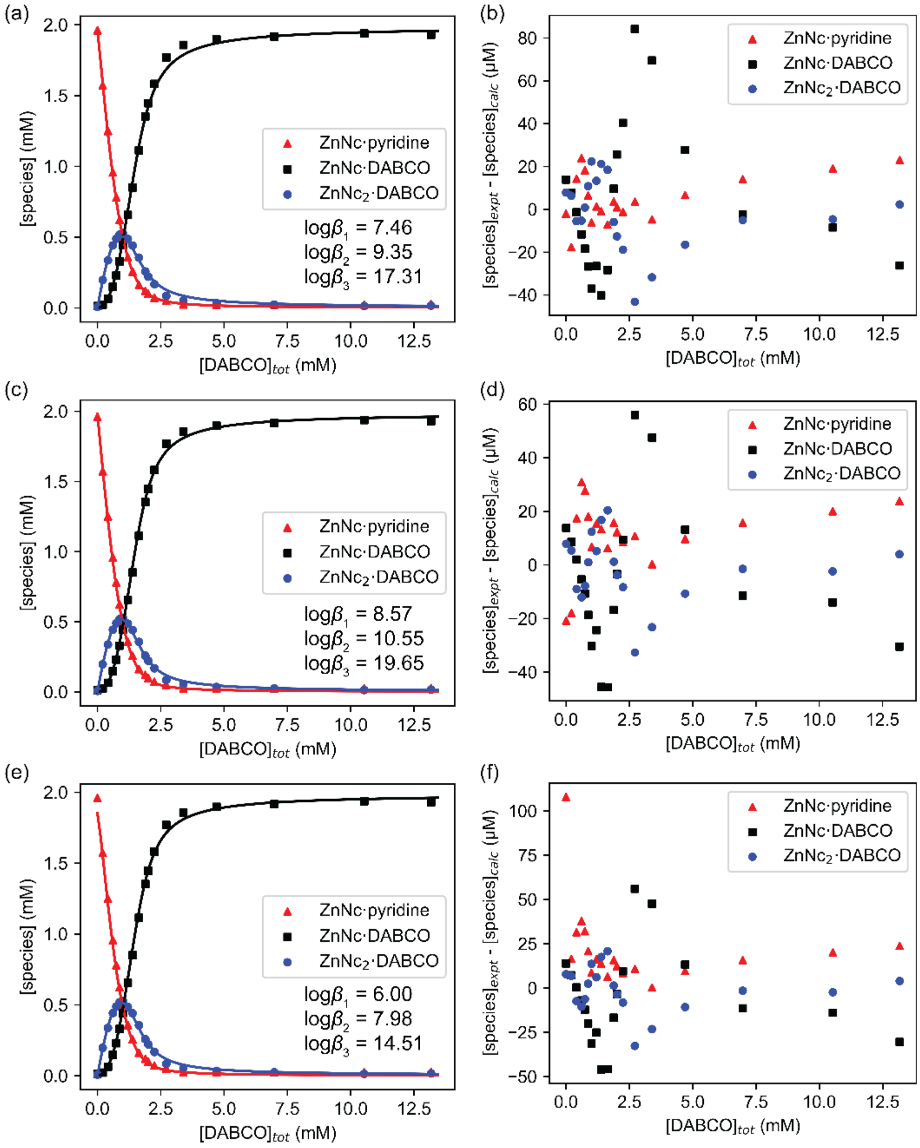

Figure S14 (left) Species concentrations determined from the titration data in Table S2 (points) and corresponding speciation calculated from the fitted equilibrium constants (lines); (right) residuals in the fit, calculated as the differences between the calculated and measured concentrations of species at each point in the titration. (a,b) are from the least-squares solution; (c,d) are the maximum likelihood estimate; (e,f) represent an example where logβ_1 (K_{py}) is set too low, causing the initial [ZnNc•pyr] to be underestimated since the model expects some free ZnNc.

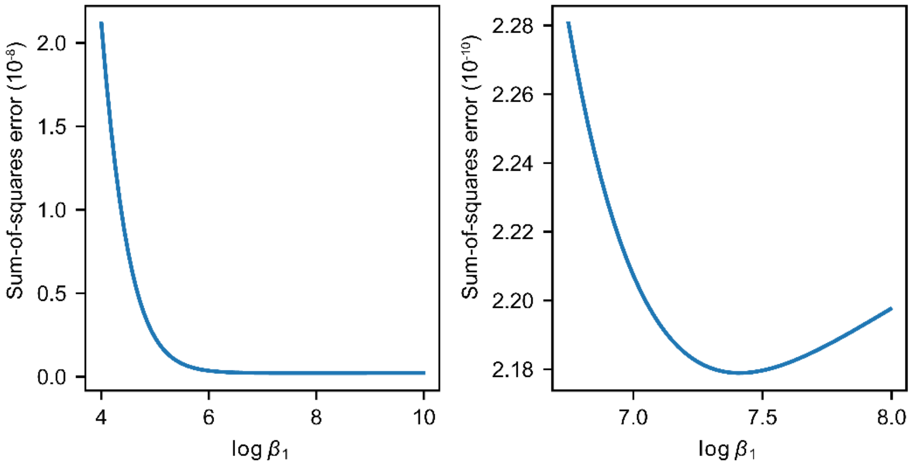

Figure S15 Effect of choice of $\log \beta_1$ on the sum-of-squares error between model and data, when $\log \beta_2 = \log \beta_1 + 1.98$ and $\log \beta_3 = 2 \log \beta_1 + 2.51$. Although the rightmost figure shows a minimum at $\log \beta_1 = 7.4$, it is important to note that these plots neglect experimental error. The plots demonstrate that when $\log \beta_1$ is greater than approx. 6-7, the fit results are generally reasonable.

We can therefore present the following conclusions:

1. The binding constant of pyridine to ZnNc is at least 10^6 M^{-1}, and therefore is at least 1 order of magnitude higher than the binding constant of pyridine to porphyrin.
2. The binding constant of DABCO to ZnNc is approximately 2 orders of magnitude higher than that of pyridine to ZnNc.
3. The maximum-likelihood estimates for the speciation constants, within a range taken as the1-sigma level of the distribution of samples, are reasonable estimates subject to the assumptions in our model, and are given in Table S3.

Table S3: Speciation constants for complexes of ZnNc, extracted from analysis of the NMR titration of DABCO into a solution of ZnNc•pyridine. The least-squares errors are approximated from the covariance matrix of the fit.

**Species**	**$\log_{10}\beta$**	
	**Least squares**	**MLE[a]**
ZnNc•pyridine	7.45 ± 0.79	$8.57^{+2.20}_{-1.22}$
ZnNc•DABCO	9.35 ± 0.79	$10.55^{+2.20}_{-1.22}$
$ZnNc_2$•DABCO	17.31 ± 1.58	$19.65^{+4.40}_{-2.44}$

a The maximum-likelihood estimate (MLE) comes from the Markov-Chain Monte Carlo experiment in Figure S13. The error parameter, assuming Gaussian errors on the experimental data, had the value $\ln \sigma = -13.11^{+0.10}_{-0.09}$, which corresponds to approximately 2×10^{-6} M.

S6. X-Ray diffraction analysis

Crystals of $(ZnNc)_2$•DABCO and ZnNc•pyridine were grown by vapour diffusion of Et_2O into a solution of the compound in toluene. The X-ray diffraction data were collected by a Bruker D8 Quest single-crystal diffractometer with the microfocus Mo source (λ=0.7107Å) and Photon II area detector. Data integration and reduction were performed by the Bruker Apex4 software. The solutions were obtained by direct methods using SHELXT followed by successive refinements using full matrix least squares method against F2 using SHELXL-2018/3.[8,9] The program OLEX2 was used was used as a graphical interface to SHELX.[10]

The ZnNc•pyridine crystals employed in this study were small and weakly diffracting. Several attempts were made to collect single crystal X-ray diffraction data with suitable quality for structural characterisation. The best attempt is reported here, where the structure crystallises in the monoclinic space group *C*2 and is heavily disordered. Despite long exposure times and a low temperature data collection only reflections were observed to approximately 1.2 Å resolution. The reflections were also broad. Accordingly several restraints and constraints (including rigid bodies) were required to facilitate modelling of the structure and only the zinc(II) centre was modelled anisotropically. Additionally there is a significant disorder component present in the structure, in which an alternative position with approximately 10 % occupancy is located only marginally displaced (but inverted) relative to the main disorder component. Only the zinc(II) centre of this low occupancy component was included in the model. Despite the limitations of the data quality the connectivity of the structure is unambiguous.

Table S4 Crystal and data refinement parameters for the X-ray studies.

Crystal data	$(ZnNc)_2$•DABCO•$(Et_2O)_2$	ZnNc•pyridine
Empirical formula	$C_{86}H_{92}N_{10}O_2Zn_2$	$C_{41}H_{35}N_5Zn$
Formula weight	1428.48	663.11
Temperature/K	120.15	120.15
Crystal system	triclinic	monoclinic
Space group	*P*-1	*C*2
a/Å	9.2544(6)	16.425(3)
b/Å	20.2072(14)	8.6410(17)
c/Å	21.2170(16)	13.023(3)
α/°	104.235(2)	90
β/°	102.409(2)	114.46(3)
γ/°	99.270(2)	90
Volume/Å^3	3658.9(4)	1682.4(7)
Z	2	2
ρ_{calc}g/cm^3	1.297	1.309
μ/mm^{-1}	0.712	0.767
F(000)	1508	692
Crystal size/mm^3	0.33 × 0.15 × 0.042	0.04 × 0.06 × 0.12
Radiation	MoKα (λ = 0.71073)	MoKα (λ = 0.71073)
2Θ range for data collection/°	4.536 to 46.714	5.098 to 34.620
Index ranges	$-10 \leq h \leq 9$, $-22 \leq k \leq 21$, $0 \leq l \leq 23$	$-13 \leq h \leq 13$, $-7 \leq k \leq 7$, $-10 \leq l \leq 10$
Reflections collected	10820	5913
Independent reflections	10820 [R_{sigma} = 0.0827]	5913 [R_{sigma} = 0.0833]
Data/restraints/parameters	10820/42/918	5913/1/106
Goodness-of-fit on F^2	1.138	1.203
Final R indexes [I>=2σ (I)]	R_1 = 0.0816, wR_2 = 0.1733	R_1 = 0.1162, wR_2 = 0.2700
Final R indexes [all data]	R_1 = 0.1812, wR_2 = 0.2538	R_1 = 0.1329, wR_2 = 0.2928
Largest diff. peak/hole / e Å^{-3}	1.17/−0.88	1.472/−0.447

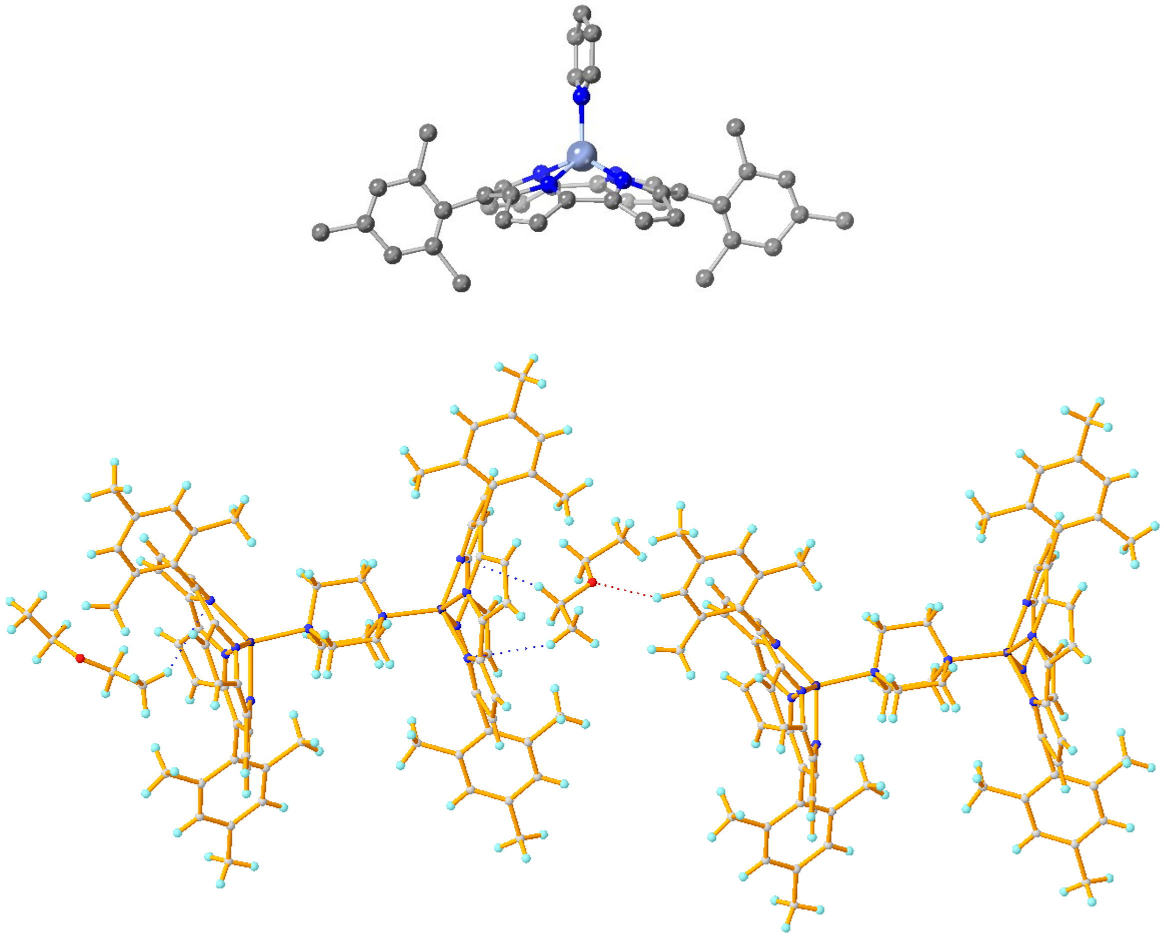

Figure S16 (top) X-ray crystal structure of ZnNc•pyridine; (bottom) packing structure of $(ZnNc)_2$•DABCO, showing interactions between norcorrole fragments and Et_2O solvent molecules.

S7. Electrochemistry

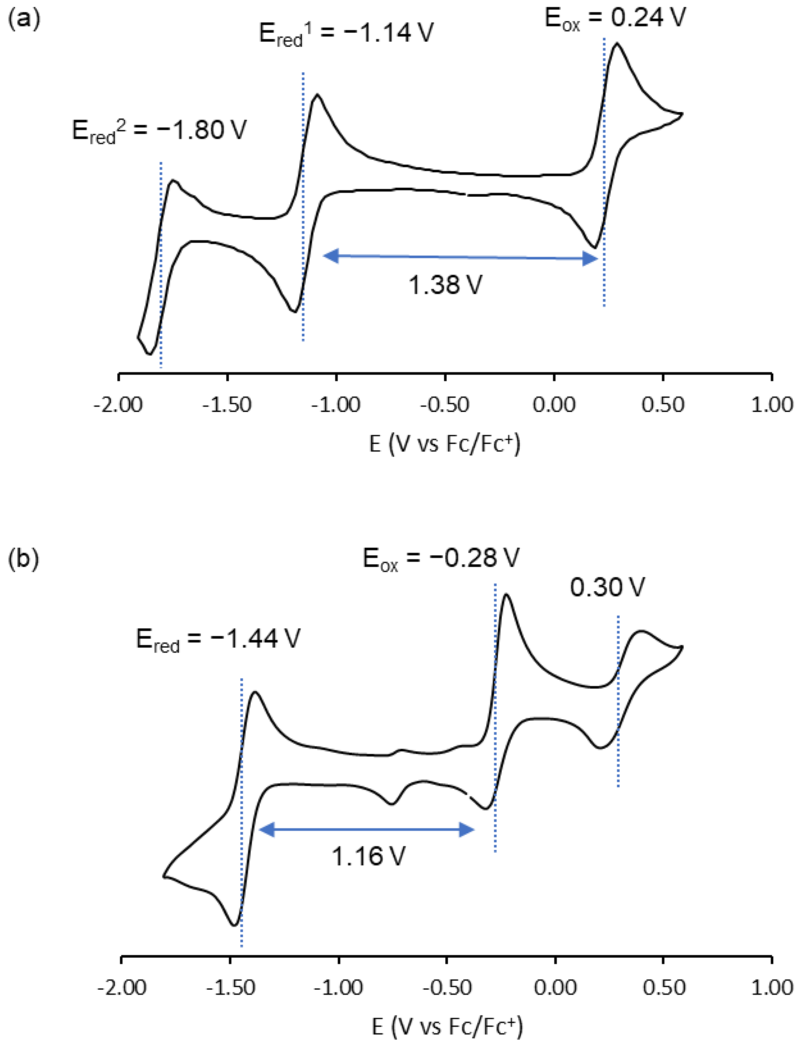

Figure S17 Cyclic voltammogram (0.1 V/s) of (a) H_2Nc and (b) ZnNc•pyridine in 0.1 M $TBAPF_6/CH_2Cl_2$ with a gold disk working electrode, Pt counter electrode, and an Ag/Ag^+ reference electrode. Ferrocene was used as an internal reference.

Table S5 First electrochemical oxidations and reductions of mesityl metallonorcorroles. Values are referenced to the potential of Fc/Fc^+.

Compound	E_{red} / V	E_{ox} / V	E_{gap} / V	Reference
ZnNc•pyridine	−1.44	−0.28	1.16	This work
H_2Nc	−1.14	0.24	1.38	This work
NiNc	−0.92	0.16	1.08	[2]
Ni oxacorrole	−1.75	0.30	2.05	[2]
PtNc	−0.82	0.12	0.94	[11]

S8. Theoretical calculations

All calculations were carried out using the Gaussian16 program.[12] The geometry and NMR chemical shielding values of ZnNc•pyridine were calculated at the B3LYP,[13,14] BLYP35,[15] BHandHLYP,[16,17] CAM-B3LYP,[18] and M06-2X[19] levels using the 6-31G* basis set.[20–23] NMR calculations used an implicit solvent model (PCM) with dichloromethane as solvent. Chemical shielding values were converted to chemical shifts according to

$$\delta(ppm) = \sigma_{TMS} - \sigma_{ZnNc}$$

where the chemical shielding of the TMS protons was calculated using the same functional and basis set. R^2 values in the tables below were calculated by fitting a straight line between the experimental chemical shifts and the DFT-predicted chemical shifts.

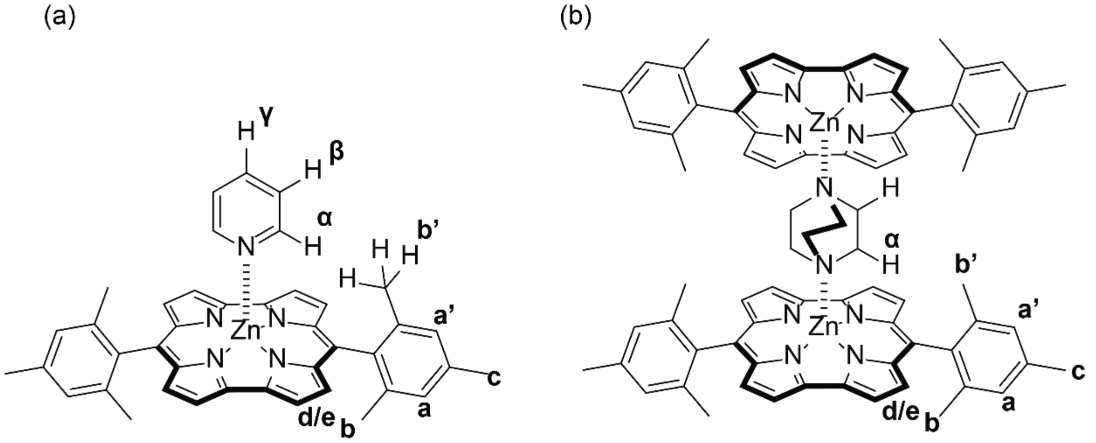

Figure S18 Labelled protons for chemical shift assignments in ZnNc•pyridine (left) and $(ZnNc)_2$•DABCO (right).

Table S6 Chemical shifts (ppm) of the hydrogen atoms in ZnNc·pyridine. See Figure S18 for atom labels. Experimental chemical shifts are based on ^1H NMR analysis of ZnNc•pyridine in CD_2Cl_2 (600 MHz, 298 K).

		ZnNc•pyridine, DFT basis set = 6-31G*						
Assignment	Exp.	B3LYP	BLYP35	BHandHLYP	CAM-B3LYP	M06-2X	B3LYP[a]	BLYP35[a]
α	13.89	14.09	13.53	13.11	12.71	13.79	14.53	13.73
β	9.31	9.24	9.16	9.08	8.84	9.73	9.56	9.40
γ	9.34	9.29	9.33	9.35	9.07	9.80	9.64	9.62
a	6.81	6.84	6.91	7.04	6.96	7.60	7.12	7.13
a′	6.34	6.28	6.43	6.61	6.60	7.11	6.46	6.60
b	4.10	4.38	4.02	3.80	3.56	4.16	4.88	4.29
d	3.93	3.80	4.04	4.45	4.71	4.84	3.82	4.13
e	3.79	3.55	3.73	4.09	4.39	4.59	3.61	3.88
c	2.02	1.97	2.01	2.10	2.09	2.19	2.14	2.17
b′	1.82	1.52	1.61	1.72	1.82	1.72	1.47	1.61
R^2	-	0.998	0.999	0.993	0.985	0.989	0.995	0.998
MAE	-	0.14	0.12	0.28	0.43	0.46	0.32	0.20
RMSE	-	0.17	0.15	0.35	0.55	0.56	0.38	0.21

[a] both optimization and NMR calculations used the 6-311G* basis set.

Table S7 Chemical shifts (ppm) of the BLYP35/6-31G* geometry of ZnNc•pyridine using different basis sets. See Figure S18 for atom labels. Experimental chemical shifts are based on ^{1}H NMR analysis of ZnNc•pyridine in CD_2Cl_2 (600 MHz, 298 K).

		ZnNc•pyridine, DFT functional = BLYP35					
Assignment	Exp.	6-311G*	6-311G**	6-311++G**	6-31G*	6-31G**	6-31+G*
α	13.89	13.64	13.66	13.93	13.53	13.61	13.71
β	9.31	9.33	9.42	9.54	9.16	9.34	9.37
γ	9.34	9.54	9.61	9.68	9.33	9.45	9.55
a	6.81	7.07	7.21	7.24	6.91	7.03	7.13
a′	6.34	6.58	6.72	6.70	6.43	6.55	6.54
b	4.10	4.14	4.11	4.22	4.02	4.02	4.09
d	3.93	4.12	4.32	4.30	4.04	4.32	4.03
e	3.79	3.87	4.07	4.04	3.73	4.00	3.85
c	2.02	2.17	2.15	2.17	2.01	2.02	2.14
b′	1.82	1.70	1.70	1.79	1.61	1.62	1.75
R^2	-	0.998	0.997	0.998	0.999	0.997	0.999
MAE	-	0.16	0.23	0.23	0.12	0.17	0.13
RMSE	-	0.18	0.27	0.27	0.15	0.21	0.16

Table S8 Chemical shifts (ppm) of the hydrogen atoms in $(ZnNc)_2$•DABCO using the 6-31G* basis set with the corresponding DFT functional. See Figure S18 for atom labels. Experimental chemical shifts are based on ^{1}H NMR analysis of $(ZnNc)_2$•DABCO in CD_2Cl_2 (600 MHz, 268 K).

		$(ZnNc)_2$•DABCO, DFT basis set = 6-31G*					
Assignment	Exp.	B3LYP	BLYP35	BHandHLYP	CAM-B3LYP	B3lYP[a]	BLYP35[a]
α	10.29	10.36	9.72	8.86	8.45	11.18	9.90
a	6.92	6.64	7.04	7.15	7.03	7.25	7.29
a′	6.42	6.05	6.52	6.70	6.65	6.54	6.70
b	4.41	4.45	4.40	4.07	3.77	5.27	4.69
d	4.09	3.38	4.13	4.58	4.70	3.92	4.28
e	3.96	3.05	3.78	4.19	4.33	3.67	3.99
c	2.13	1.70	2.07	2.15	2.11	2.20	2.24
b′	2.06	1.58	1.95	2.03	2.06	1.88	1.98
R^2	-	0.991	0.994	0.962	0.936	0.987	0.992
MAE	-	0.41	0.15	0.38	0.48	0.36	0.22
RMSE	-	0.50	0.23	0.57	0.74	0.48	0.25

[a] both optimization and NMR calculations used the 6-311G* basis set.

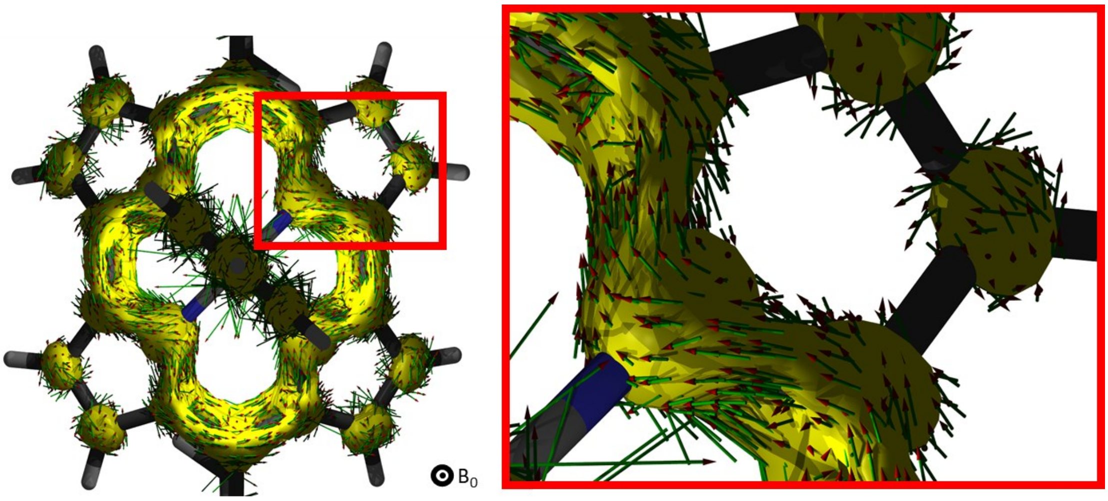

Figure S19 AICD plot (isovalue of 0.14) of ZnNc•pyridine at the B3LYP/6-31G* level, with the applied magnetic field vector pointing towards the viewer. The arrows are pointing anticlockwise.

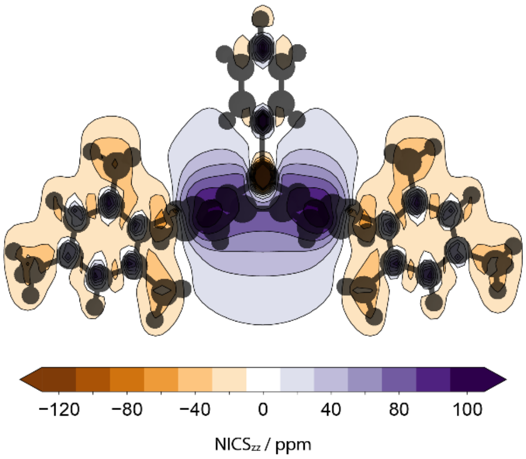

Figure S20 NICS plot of ZnNc•pyridine complex, overlayed with the structure of ZnNc•pyridine which was optimised at the BLYP35/6-31G* level.

S9. Ring current analysis

We used our previously-described method based on ring-current geometric factors[24,25] to estimate the ring current strength in norcorrole. The ring current geometric factors (RCGFs) were determined from calculated (BLYP35/6-31G*) geometries of ZnNc•pyridine, ZnNc•DABCO and $(ZnNc)_2$•DABCO, based on π-conjugated paths which were equally-weighted at each bifurcation point, and where the pi-conjugation pathway was simulated with an 0.7 Å offset above and below the plane.

Chemical shift differences attributable to norcorrole (anti)aromaticity were determined as follows:

$$\Delta\delta = \delta_{obs} - \delta_{ref}$$

where δ_{ref} is from an analogous compound without antiaromatic influences. We determined $\Delta\delta$ values for pyridine, DABCO, and the mesityl side groups, and used the NMR spectra of pyridine, DABCO, and mesitylene, respectively, for δ_{ref}.

Having obtained both $\Delta\delta$ and RCGF values, we could extract the ring current susceptibility $\frac{I}{B}$ from:

$$\Delta\delta = \frac{I}{B}\text{RCGF}$$

where RCGF has units (μT/nA), $\Delta\delta$ has units (ppm), and $\frac{I}{B}$ accordingly has units (nA/T).

S10. References

(1) Yonezawa, T.; Shafie, S. A.; Hiroto, S.; Shinokubo, H. Shaping Antiaromatic π-Systems by Metalation: Synthesis of a Bowl-Shaped Antiaromatic Palladium Norcorrole. *Angew. Chem. Int. Ed.* **2017**, *56* (39), 11822–11825. https://doi.org/10.1002/anie.201706134.

(2) Ito, T.; Hayashi, Y.; Shimizu, S.; Shin, J.-Y.; Kobayashi, N.; Shinokubo, H. Gram-Scale Synthesis of Nickel(II) Norcorrole: The Smallest Antiaromatic Porphyrinoid. *Angew. Chem. Int. Ed.* **2012**, *51* (34), 8542–8545. https://doi.org/10.1002/anie.201204395.

(3) 3 Physical/Chemical Models. In *Practical Data Analysis in Chemistry*; Maeder, M., Neuhold, Y.-M., Eds.; Data Handling in Science and Technology; Elsevier, 2007; Vol. 26, pp 29–100. https://doi.org/10.1016/S0922-3487(07)80006-2.

(4) Virtanen, P.; Gommers, R.; Oliphant, T. E.; Haberland, M.; Reddy, T.; Cournapeau, D.; Burovski, E.; Peterson, P.; Weckesser, W.; Bright, J.; van der Walt, S. J.; Brett, M.; Wilson, J.; Millman, K. J.; Mayorov, N.; Nelson, A. R. J.; Jones, E.; Kern, R.; Larson, E.; Carey, C. J.; Polat, İ.; Feng, Y.; Moore, E. W.; VanderPlas, J.; Laxalde, D.; Perktold, J.; Cimrman, R.; Henriksen, I.; Quintero, E. A.; Harris, C. R.; Archibald, A. M.; Ribeiro, A. H.; Pedregosa, F.; van Mulbregt, P. SciPy 1.0: Fundamental Algorithms for Scientific Computing in Python. *Nat. Methods* **2020**, *17* (3), 261–272. https://doi.org/10.1038/s41592-019-0686-2.

(5) Newville, M.; Otten, R.; Nelson, A.; Stensitzki, T.; Ingargiola, A.; Allan, D.; Fox, A.; Carter, F.; Michał; Osborn, R.; Pustakhod, D.; lneuhaus; Weigand, S.; Aristov, A.; Glenn; Deil, C.; mgunyho; Mark; Hansen, A. L. R.; Pasquevich, G.; Foks, L.; Zobrist, N.; Frost, O.; Stuermer; azelcer; Polloreno, A.; Persaud, A.; Nielsen, J. H.; Pompili, M.; Eendebak, P. Lmfit/Lmfit-Py: 1.2.2, 2023. https://doi.org/10.5281/zenodo.8145703.

(6) Foreman-Mackey, D.; Hogg, D. W.; Lang, D.; Goodman, J. Emcee: The MCMC Hammer. *Publ. Astron. Soc. Pac.* **2013**, *125* (925), 306–312. https://doi.org/10.1086/670067.

(7) Foreman-Mackey, D. Corner.Py: Scatterplot Matrices in Python. *J. Open Source Softw.* **2016**, *1* (2), 24. https://doi.org/10.21105/joss.00024.

(8) Sheldrick, G. M. A Short History of SHELX. *Acta Crystallogr. A* **2008**, *64* (1), 112–122. https://doi.org/10.1107/S0108767307043930.

(9) Sheldrick, G. M. Crystal Structure Refinement with SHELXL. *Acta Crystallogr. Sect. C Struct. Chem.* **2015**, *71* (1), 3–8. https://doi.org/10.1107/S2053229614024218.

(10) Dolomanov, O. V.; Bourhis, L. J.; Gildea, R. J.; Howard, J. A. K.; Puschmann, H. OLEX2: A Complete Structure Solution, Refinement and Analysis Program. *J. Appl. Crystallogr.* **2009**, *42* (2), 339–341. https://doi.org/10.1107/S0021889808042726.
(11) Kawashima, H.; Fukui, N.; Phung, Q. M.; Yanai, T.; Shinokubo, H. Planarization of a Bowl-Shaped Molecule by Triple-Decker Stacking. *Cell Rep. Phys. Sci.* **2022**, *3* (9), 101045. https://doi.org/10.1016/j.xcrp.2022.101045.
(12) Frisch, M. J.; Trucks, G. W.; Schlegel, H. B.; Scuseria, G. E.; Robb, M. A.; Cheeseman, J. R.; Scalmani, G.; Barone, V.; Petersson, G. A.; Nakatsuji, H.; Li, X.; Caricato, M.; Marenich, A. V.; Bloino, J.; Janesko, B. G.; Gomperts, R.; Mennucci, B.; Hratchian, H. P.; Ortiz, J. V.; Izmaylov, A. F.; Sonnenberg, J. L.; Williams; Ding, F.; Lipparini, F.; Egidi, F.; Goings, J.; Peng, B.; Petrone, A.; Henderson, T.; Ranasinghe, D.; Zakrzewski, V. G.; Gao, J.; Rega, N.; Zheng, G.; Liang, W.; Hada, M.; Ehara, M.; Toyota, K.; Fukuda, R.; Hasegawa, J.; Ishida, M.; Nakajima, T.; Honda, Y.; Kitao, O.; Nakai, H.; Vreven, T.; Throssell, K.; Montgomery Jr., J. A.; Peralta, J. E.; Ogliaro, F.; Bearpark, M. J.; Heyd, J. J.; Brothers, E. N.; Kudin, K. N.; Staroverov, V. N.; Keith, T. A.; Kobayashi, R.; Normand, J.; Raghavachari, K.; Rendell, A. P.; Burant, J. C.; Iyengar, S. S.; Tomasi, J.; Cossi, M.; Millam, J. M.; Klene, M.; Adamo, C.; Cammi, R.; Ochterski, J. W.; Martin, R. L.; Morokuma, K.; Farkas, O.; Foresman, J. B.; Fox, D. J. Gaussian 16 Rev. C.01, 2016.
(13) Becke, A. D. Density-functional Thermochemistry. III. The Role of Exact Exchange. *J. Chem. Phys.* **1993**, *98* (7), 5648–5652. https://doi.org/10.1063/1.464913.
(14) Stephens, P. J.; Devlin, F. J.; Chabalowski, C. F.; Frisch, M. J. Ab Initio Calculation of Vibrational Absorption and Circular Dichroism Spectra Using Density Functional Force Fields. *J. Phys. Chem.* **1994**, *98* (45), 11623–11627. https://doi.org/10.1021/j100096a001.
(15) Renz, M.; Theilacker, K.; Lambert, C.; Kaupp, M. A Reliable Quantum-Chemical Protocol for the Characterization of Organic Mixed-Valence Compounds. *J. Am. Chem. Soc.* **2009**, *131* (44), 16292–16302. https://doi.org/10.1021/ja9070859.
(16) Becke, A. D. A New Mixing of Hartree–Fock and Local Density-functional Theories. *J. Chem. Phys.* **1993**, *98* (2), 1372–1377. https://doi.org/10.1063/1.464304.
(17) Adamo, C.; Barone, V. Toward Reliable Adiabatic Connection Models Free from Adjustable Parameters. *Chem. Phys. Lett.* **1997**, *274* (1), 242–250. https://doi.org/10.1016/S0009-2614(97)00651-9.
(18) Yanai, T.; Tew, D. P.; Handy, N. C. A New Hybrid Exchange–Correlation Functional Using the Coulomb-Attenuating Method (CAM-B3LYP). *Chem. Phys. Lett.* **2004**, *393* (1), 51–57. https://doi.org/10.1016/j.cplett.2004.06.011.
(19) Zhao, Y.; Truhlar, D. G. The M06 Suite of Density Functionals for Main Group Thermochemistry, Thermochemical Kinetics, Noncovalent Interactions, Excited States, and Transition Elements: Two New Functionals and Systematic Testing of Four M06-Class Functionals and 12 Other Functionals. *Theor. Chem. Acc.* **2008**, *120* (1), 215–241. https://doi.org/10.1007/s00214-007-0310-x.
(20) Ditchfield, R.; Hehre, W. J.; Pople, J. A. Self-Consistent Molecular-Orbital Methods. IX. An Extended Gaussian-Type Basis for Molecular-Orbital Studies of Organic Molecules. *J. Chem. Phys.* **1971**, *54* (2), 724–728. https://doi.org/10.1063/1.1674902.
(21) Hehre, W. J.; Ditchfield, R.; Pople, J. A. Self—Consistent Molecular Orbital Methods. XII. Further Extensions of Gaussian—Type Basis Sets for Use in Molecular Orbital Studies of Organic Molecules. *J. Chem. Phys.* **1972**, *56* (5), 2257–2261. https://doi.org/10.1063/1.1677527.
(22) Hariharan, P. C.; Pople, J. A. The Influence of Polarization Functions on Molecular Orbital Hydrogenation Energies. *Theor. Chim. Acta* **1973**, *28* (3), 213–222. https://doi.org/10.1007/BF00533485.
(23) Rassolov, V. A.; Pople, J. A.; Ratner, M. A.; Windus, T. L. 6-31G* Basis Set for Atoms K through Zn. *J. Chem. Phys.* **1998**, *109* (4), 1223–1229. https://doi.org/10.1063/1.476673.
(24) Jirásek, M.; Anderson, H. L.; Peeks, M. D. From Macrocycles to Quantum Rings: Does Aromaticity Have a Size Limit? *Acc. Chem. Res.* **2021**, *54* (16), 3241–3251. https://doi.org/10.1021/acs.accounts.1c00323.
(25) Bradley, D.; Jirásek, M.; Anderson, H. L.; Peeks, M. D. Disentangling Global and Local Ring Currents. *Chem. Sci.* **2023**, *14*, 1762–1768. https://doi.org/10.1039/D2SC05923A.
